# Prognostic Impact of RTK–RAS Alterations in FOLFOX-Treated Early-Onset Colorectal Cancer Revealed by Artificial Intelligence-Driven Precision Oncology

**DOI:** 10.3390/cancers18020239

**Published:** 2026-01-13

**Authors:** Fernando C. Diaz, Brigette Waldrup, Francisco G. Carranza, Sophia Manjarrez, Enrique Velazquez-Villarreal

**Affiliations:** 1 Lineberger Comprehensive Cancer Center, University of North Carolina, Chapel Hill, NC 27514, USA; 2 Department of Integrative Translational Sciences, Beckman Research Institute, City of Hope, Duarte, CA 91010, USA; 3 City of Hope Comprehensive Cancer Center, Duarte, CA 91010, USA

**Keywords:** early-onset colorectal cancer, RTK–RAS signaling pathway, genetic epidemiology, cancer epidemiology, cancer incidence, FOLFOX chemotherapy, artificial intelligence, AI-agents, biomarkers

## Abstract

Background/Objectives: Early-onset colorectal cancer (EOCRC; diagnosed before age 50) is rising at an accelerated rate, with a disproportionate impact on underserved populations. While alterations in the receptor tyrosine kinase–RAS (RTK–RAS) signaling pathway play a fundamental role in colorectal cancer (CRC) biology, their prognostic significance in the setting of FOLFOX chemotherapy—particularly across different age groups and ancestral backgrounds—remains insufficiently characterized. We sought to characterize age-, ancestry-, and treatment-specific associations between RTK–RAS alterations and clinical outcomes using an AI-enabled precision oncology framework. Methods: We analyzed 2515 CRC cases, including 266 Hispanic/Latino (H/L) and 2249 non-Hispanic White (NHW) patients, stratified by age at onset, ancestry, and FOLFOX treatment status. Mutation frequencies were assessed using Fisher’s exact and chi-square tests, while overall survival was analyzed with Kaplan–Meier methods. The AI-HOPE and AI-HOPE–RTK–RAS conversational artificial intelligence platforms were used to integrate clinical, genomic, and treatment data via multi-parameter, natural language–based queries. Results: In early-onset Hispanic/Latino patients, ERBB2 and NF1 mutations occurred at significantly lower frequencies in FOLFOX-treated cases compared with untreated cases (*p* = 0.01 for both). In late-onset H/L patients, NTRK2 mutations were depleted in FOLFOX-treated tumors (*p* = 0.04). In untreated early-onset H/L patients, MAPK3 and NF1 mutations were enriched relative to NHW counterparts. Among early-onset NHW patients, IGF1R and ERRFI1 mutations were less frequent with FOLFOX exposure, while multiple RTK–RAS genes were reduced in FOLFOX-treated late-onset NHW patients. Survival analyses revealed worse overall survival in FOLFOX-untreated early-onset NHW patients with RTK–RAS alterations (*p* = 0.029), but improved survival in FOLFOX-treated late-onset NHW patients (*p* = 0.048). Conclusions: RTK–RAS pathway alterations demonstrate strong age-, ancestry-, and treatment-specific prognostic effects and may serve as precision biomarkers of differential chemotherapy response. AI-enabled analytics substantially accelerated integrative biomarker discovery, supporting their utility for advancing precision oncology in EOCRC.

## Simple Summary

Colorectal cancer diagnosed before age 50, known as early-onset colorectal cancer, is becoming more common, especially in diverse and underserved populations. Changes in the RTK–RAS signaling pathway play an important role in colorectal cancer, but how these changes affect patient outcomes can depend on age, ancestry, and treatment. In this study, we analyzed clinical and genomic data from more than 2500 colorectal cancer patients using an artificial intelligence (AI)-enabled precision oncology platform. We found that RTK–RAS pathway alterations were associated with different survival outcomes depending on whether patients received FOLFOX chemotherapy and on their ancestry. Specifically, these alterations were associated with poorer survival among early-onset non-Hispanic White patients who were not treated with FOLFOX, whereas they correlated with better survival in late-onset non-Hispanic White patients who received FOLFOX. Together, these results underscore the critical need to integrate age at diagnosis, ancestry, and treatment context when applying genomic biomarkers to inform colorectal cancer management.

## 1. Introduction

Colorectal cancer (CRC) remains a major global cause of cancer morbidity and mortality, and contemporary management increasingly depends on molecular stratification to guide systemic therapy choices across the disease continuum [1,2,3,4]. In parallel with advances in screening and treatment, a concerning epidemiologic shift has emerged: early-onset colorectal cancer (EOCRC), defined as diagnosis before age 50, is rising in multiple populations, expanding the clinical urgency to understand whether tumor biology, and treatment response, differs by age at onset and ancestry [1,3]. Because EOCRC patients frequently present with distinct clinical trajectories and longer life-years at risk from treatment-related toxicities, identifying biomarkers that inform chemotherapy benefit versus harm is particularly important in this setting [1,2,3].

A central axis in CRC oncogenesis and therapy selection is the receptor tyrosine kinase–RAS signaling cascade (RTK–RAS), encompassing upstream receptor alterations (e.g., *ERBB* family, *IGF1R*, *KIT*), negative regulators (e.g., *NF1*, *ERRFI1*), and downstream RAF-MAPK effectors. Alterations in *KRAS/NRAS/BRAF* are routinely evaluated because RAS activation predicts lack of benefit from anti-EGFR monoclonal antibodies and strongly influences targeted-therapy sequencing in metastatic CRC [1,4,5,6,7,8,9,10,11]. Large randomized trials established the clinical impact of RAS status when combining EGFR inhibitors with oxaliplatin-based chemotherapy (FOLFOX), demonstrating improved outcomes in RAS-wild-type disease and potential detriment in RAS-mutant subgroups [6,7,12,13,14]. More broadly, RTK–RAS pathway dependencies and resistance circuits remain a major focus of precision medicine efforts, particularly as therapeutic options for RAS-mutant cancers expand and combination strategies evolve [3,15,16,17,18,19].

Despite this progress, the prognostic and predictive implications of RTK–RAS alterations in patients receiving standard cytotoxic regimens, especially FOLFOX, remain incompletely defined [20,21,22,23,24,25]. Prior studies evaluating KRAS and related markers in the context of oxaliplatin/fluoropyrimidine therapy have reported heterogeneous results across settings, including adjuvant and metastatic disease, and across endpoints such as disease-free survival, progression-free survival, and overall survival [26,27,28,29,30,31]. Mechanistic work further suggests that oxaliplatin/5-FU exposure may interact with *KRAS*-driven signaling to shape phenotypes relevant to resistance and tumor aggressiveness, underscoring the plausibility of gene-treatment interactions within the RTK–RAS axis [19,25,32]. However, most available evidence has not been designed to resolve whether RTK–RAS alterations exert age-at-onset-specific or ancestry-associated effects on outcomes after FOLFOX exposure, an important gap given the rising burden of EOCRC and the need to advance equitable precision oncology [1,3,4].

To address these limitations, we performed an integrative clinical-genomic analysis of 2515 CRC cases (Hispanic/Latino [H/L] = 266; non-Hispanic White [NHW] = 2249) stratified by ancestry, age at onset (EOCRC vs. late-onset CRC [LOCRC]), and FOLFOX treatment status [33,34,35]. Using Fisher’s exact and chi-square tests for alteration frequency comparisons and Kaplan–Meier survival analyses for outcome associations, we interrogated RTK–RAS pathway alterations [36,37] as candidate biomarkers of differential chemotherapy response. We additionally leveraged AI-HOPE [38] and the pathway-specialized AI-HOPE–RTK–RAS [39] conversational artificial intelligence framework to accelerate multi-parameter integration of clinical, genomic, and treatment-level variables through natural language-driven analytic queries, enabling rapid iteration across clinically relevant subgroup definitions.

Here, we show that RTK–RAS alterations exhibit pronounced age-, ancestry-, and treatment-specific patterns, including differential enrichment of *ERBB2*, *NF1*, *NTRK2*, *IGF1R*, and *ERRFI1* by FOLFOX exposure status across EO and LO strata, and divergent survival associations in key subgroups [40,41,42,43,44,45]. Collectively, these findings support RTK–RAS alterations as potential precision biomarkers of chemotherapy response heterogeneity and illustrate how AI-enabled analytics can help scale rigorous, subgroup-aware biomarker discovery for EOCRC populations [46,47,48,49,50].

Although RTK–RAS signaling has been extensively studied in colorectal cancer, prior work has largely focused on its role in predicting response to targeted anti-EGFR therapies, with far less clarity regarding its prognostic and predictive relevance in the context of standard cytotoxic regimens such as FOLFOX. Existing studies have often yielded inconsistent results, have been limited to single genes (e.g., *KRAS* or *BRAF*), and rarely account for critical sources of heterogeneity such as age at onset or ancestry. As a result, it remains unclear whether RTK–RAS alterations confer differential survival effects in early-onset versus late-onset disease or across populations that have been historically underrepresented in genomic studies. Moreover, conventional analytic approaches are typically constrained by rigid cohort definitions and manual data handling, limiting their ability to systematically explore high-dimensional gene–treatment–demographic interactions. In this context, AI-driven analytical frameworks offer a distinct advantage by enabling scalable, multi-parameter integration of clinical, genomic, and treatment data, rapid iteration across complex subgroup definitions, and hypothesis testing that more closely reflects real-world clinical heterogeneity. This approach (Figure S1) allows previously tangled, context-dependent RTK–RAS associations to be identified with greater resolution, directly addressing key gaps in prior precision oncology research.

## 2. Materials and Methods

### 2.1. Study Design and Data Acquisition

We performed a retrospective, multi-cohort precision oncology analysis integrating clinical, genomic, and treatment data from publicly available colorectal cancer (CRC) datasets accessed through the cBioPortal for Cancer Genomics [51]. Data were obtained from three independent CRC cohorts selected for their comprehensive somatic mutation profiling and detailed therapeutic annotations: The colorectal cancer cohorts from The Cancer Genome Atlas (TCGA, PanCancer Atlas) [52], MSK-CHORD [53], and the AACR Project GENIE Biopharma Collaborative (BPC) [54].

Patients were considered eligible if they had histologically confirmed adenocarcinoma of the colon, rectum, or colorectum and if tumor sequencing data were available from either primary or metastatic specimens. To prevent sample-level duplication, a single representative tumor sample per patient was retained based on completeness of clinical and treatment annotation. All datasets were fully de-identified and publicly accessible.

### 2.2. Ancestry Assignment and Age-at-Onset Stratification

Patient ancestry was determined using self-reported ethnicity annotations when available. Individuals were classified as H/L if designated as “Hispanic or Latino,” “Latino,” “Hispanic, NOS,” or equivalent categories. For cases lacking explicit ethnicity data, validated surname-based algorithms were applied to infer Hispanic origin. Patients not meeting H/L criteria were categorized as NHW and served as the comparator population. In cases where self-reported ethnicity or structured ancestry annotations were unavailable (Table 1), surname-based ancestry inference was applied as a complementary method to classify Hispanic/Latino versus non-Hispanic White status.

Clinical metadata were used to determine age at diagnosis, with early-onset colorectal cancer (EOCRC) defined as diagnosis before 50 years of age and late-onset colorectal cancer (LOCRC) defined as diagnosis at 50 years or older. All subsequent analyses were systematically stratified by ancestry (H/L vs. NHW) and by age-at-onset category (EOCRC vs. LOCRC).

### 2.3. Chemotherapy Exposure Classification

Treatment histories were systematically reviewed to identify exposure to FOLFOX chemotherapy, defined as administration of fluorouracil (5-FU), leucovorin, and oxaliplatin either concurrently or as part of a documented treatment sequence consistent with standard clinical practice. Patients were classified as FOLFOX-treated if all three agents were recorded with overlapping or sequential treatment dates indicative of a complete regimen.

Patients lacking documentation of one or more FOLFOX components were classified as non-FOLFOX-treated. Treatment classification was performed prior to molecular stratification to avoid bias in subgroup assignment. FOLFOX treatment status was determined based on available clinical annotations within each dataset and reflected documented exposure to oxaliplatin-based chemotherapy. Information regarding treatment timing, duration, or line of therapy (adjuvant versus metastatic setting) was not consistently available across cohorts and therefore could not be uniformly incorporated into the analyses.

### 2.4. RTK–RAS Pathway Gene Curation and Alteration Definition

The RTK–RAS signaling pathway was defined using a curated gene list derived from established oncogenic signaling frameworks and CRC-focused literature. The pathway included receptor tyrosine kinases (e.g., *ERBB2, ERBB3, IGF1R, KIT, RET, ALK, NTRK2, FLT3*), negative regulators (e.g., *NF1, ERRFI1*), and downstream effectors within the RAF-MAPK cascade (e.g., *ARAF, RAF1, MAPK3*) (Tables S1–S13).

Somatic alterations were obtained from cBioPortal mutation calls, retaining only protein-altering variants such as missense, nonsense, frameshift insertions or deletions, splice-site, and start codon mutations. RTK–RAS pathway status was then defined in a binary manner, whereby patients were classified as “altered” if at least one qualifying mutation was identified in any gene within the pathway.

Missing data handling for microsatellite instability (MSI) and tumor mutational burden (TMB) was addressed using an exclusion-based approach. No imputation was performed for missing MSI or TMB values. Instead, cases lacking MSI or TMB annotations were excluded only from analyses that explicitly required these variables, while remaining eligible for all other clinical, genomic, and survival analyses. This strategy was implemented to minimize potential bias associated with imputation of molecular features and to preserve the largest possible sample size for analyses not dependent on MSI or TMB status.

### 2.5. Data Analysis and Statistics

Mutation frequencies were evaluated across ancestry, age-at-onset, and treatment-defined subgroups using Fisher’s exact or chi-square tests, selected according to cell size. Continuous clinical variables were analyzed with nonparametric methods. Overall survival (OS) was calculated from the date of diagnosis to death or last follow-up, with survival curves estimated using the Kaplan–Meier approach and compared via the log-rank test. A two-sided *p*-value < 0.05 was considered statistically significant, and all analyses were conducted using R software (version 4.3.2). The analyses presented in this study were conducted primarily using data derived from a single dominant source (Table S14), thereby minimizing technical variability associated with cross-platform or multi-batch integration. As a result, additional normalization or batch-effect correction procedures were not applied. Implementing such adjustments in this context was not recommended, as they were unlikely to provide meaningful benefit and could introduce unintended bias or distort true biological signals. This approach was chosen to preserve the integrity of the original clinical and genomic measurements while ensuring consistency across all analyses.

### 2.6. AI-Enabled Integrative Analysis Using AI-HOPE and AI-HOPE–RTK–RAS

To support high-dimensional data integration and enable systematic evaluation of ancestry-, age-, and treatment-specific molecular patterns, we utilized the AI-HOPE [38] conversational artificial intelligence framework along with its pathway-focused module, AI-HOPE–RTK–RAS [39]. These AI agents integrate structured clinical information, genomic alteration profiles, and treatment annotations, allowing complex multi-parameter analyses to be performed through natural language–based queries.

The AI platforms were first used to perform an exploratory post-hoc scan of the combined CRC cohorts, prioritizing clinically actionable questions such as: (i) whether RTK–RAS alterations were differentially enriched by FOLFOX exposure within EOCRC and LOCRC subgroups; (ii) whether ancestry-specific mutation patterns emerged within treatment-defined strata; and (iii) whether RTK–RAS alteration status modified overall survival in a treatment-dependent manner.

After this AI-driven prioritization step, AI-HOPE-RTK–RAS [39] was applied to automatically define patient cohorts based on combined criteria—including ancestry, age at onset, treatment exposure, and pathway alteration status—generate subgroup-specific mutation frequency tables, and stratify cohorts for survival analyses. The conversational interface allowed iterative adjustment of analytical parameters, reduced manual data processing, and streamlined the progression from hypothesis generation to statistically validated inference.

## 3. Results

### 3.1. Cohort Composition and Baseline Characteristics

The study population consisted of 2515 CRC cases with baseline demographic, clinical, and molecular features summarized in Table 1. All cases included primary tumor sequencing data and were eligible for stratification by age at onset, ancestry, and FOLFOX treatment exposure.

Sex distribution was similar between ancestry groups, showing a slight predominance of males in both cohorts (59.4% in H/L and 56.3% in NHW). Tumor types were also comparably distributed, with colon adenocarcinoma being the most common diagnosis in both populations (61.7% in H/L and 59.0% in NHW), followed by rectal adenocarcinoma and colorectal adenocarcinoma not otherwise specified.

At diagnosis, most patients in both groups had stage I–III disease, comprising 58.6% of Hispanic/Latino and 55.0% of non-Hispanic White cases. Stage IV disease was present in 40.6% of Hispanic/Latino patients and 44.7% of non-Hispanic White patients, with very little missing staging information in either cohort. Microsatellite status was largely microsatellite stable (MSS); however, MSS tumors were less frequent in Hispanic/Latino patients (75.2%) than in non-Hispanic White patients (86.3%), and a greater proportion of missing MSI data was observed in the Hispanic/Latino cohort.

Distinct patterns became evident when patients were jointly stratified by age at diagnosis and FOLFOX treatment status. Early-onset colorectal cancer (EOCRC; <50 years) treated with FOLFOX accounted for a greater proportion of the Hispanic/Latino cohort (27.4%) than the non-Hispanic White cohort (16.7%). In contrast, late-onset colorectal cancer (LOCRC; ≥50 years) treated with FOLFOX was more common among non-Hispanic White patients (40.9%) than Hispanic/Latino patients (34.2%). Additionally, Hispanic/Latino patients had a higher proportion of EOCRC cases that did not receive FOLFOX (19.5% vs. 13.4% in non-Hispanic White patients), while untreated LOCRC cases were more frequently observed in the non-Hispanic White group.

As anticipated, ethnicity annotations showed complete distinction between the two cohorts. All non-Hispanic White patients were classified as non-Spanish/non-Hispanic, whereas Hispanic/Latino patients were predominantly annotated as Spanish/Hispanic not otherwise specified (86.5%), followed by individuals of Mexican or Chicano origin (11.3%), with only minimal representation from other Hispanic/Latino subgroups.

### 3.2. Age-, Ancestry-, and Treatment-Specific Genomic Landscapes

#### 3.2.1. RTK–RAS-Related Clinical and Genomic Features in H/L Patients

Among H/L patients, age at diagnosis and chemotherapy exposure were associated with measurable differences in both clinical and molecular features (Table 2a). In EOCRC, individuals treated with FOLFOX were diagnosed at a slightly older age than those not receiving FOLFOX, although this difference did not reach statistical significance. In contrast, among LOCRC H/L patients, FOLFOX-treated individuals were diagnosed at a significantly younger age than their untreated counterparts.

Global genomic metrics were largely comparable across treatment groups in EOCRC H/L patients, including mutation burden, tumor mutational burden (TMB), and fraction of genome altered (FGA). However, in LOCRC H/L disease, non-FOLFOX-treated patients exhibited significantly higher TMB compared with those receiving FOLFOX, while mutation counts and FGA remained similar.

Despite these overall similarities, treatment-associated differences emerged at the gene level. In EOCRC H/L patients, *ERBB2* and *NF1* mutations were significantly less frequent among those treated with FOLFOX compared with untreated patients. In LOCRC H/L patients, *NTRK2* mutations were observed exclusively in the non-FOLFOX-treated group, indicating a treatment-dependent depletion of this alteration in exposed cases.

#### 3.2.2. Genomic Stratification of NHW Patients by Age and FOLFOX Exposure

Analysis of NHW patients revealed more pronounced treatment-associated genomic differences, particularly in LOCRC disease (Table 2b). Among EOCRC NHW patients, age at diagnosis and global genomic metrics, including mutation count, TMB, and FGA, did not differ significantly by FOLFOX treatment status.

In contrast, LOCRC NHW patients receiving FOLFOX were diagnosed at significantly younger ages than untreated patients and exhibited lower mutation counts and TMB. Multiple RTK–RAS pathway genes showed significantly reduced mutation frequencies in FOLFOX-treated LOCRC NHW patients, including *ERBB3, KIT, RET, ALK, FLT3, ARAF,* and *RAF1*. *ERRFI1* mutations were significantly less frequent in FOLFOX-treated EOCRC NHW patients, while IGF1R mutations were less common among FOLFOX-treated EOCRC NHW patients compared with untreated cases. These findings indicate a broad, treatment-associated shift in RTK–RAS alteration profiles in NHW CRC, most evident in LOCRC disease.

#### 3.2.3. Ancestry-Specific Differences in EOCRC

Direct comparison of EOCRC H/L and NHW patients highlighted ancestry-associated differences that were modified by treatment exposure (Table 2c). Among FOLFOX-treated EOCRC patients, age at diagnosis, mutation burden, TMB, and FGA were similar between ancestry groups. However, among EOCRC patients not treated with FOLFOX, H/L individuals were diagnosed at significantly younger ages than NHW patients.

At the gene level, *ERBB2* mutations were significantly more frequent in untreated EOCRC H/L patients compared with untreated NHW patients. Additionally, *MAPK3* and *NF1* mutations were enriched in untreated EOCRC H/L patients relative to their NHW counterparts, whereas no significant ancestry-related differences were observed among FOLFOX-treated EOCRC patients.

#### 3.2.4. Key Findings

Across ancestry and age-at-onset strata, RTK–RAS genomic landscapes demonstrated clear treatment-associated heterogeneity. In Hispanic/Latino patients, FOLFOX exposure was linked to selective depletion of specific RTK–RAS alterations, including *ERBB2* and *NF1* in EOCRC and *NTRK2* in LOCRC, with minimal impact on global genomic metrics. In contrast, non-Hispanic White patients, particularly those with LOCRC, exhibited broader treatment-associated reductions in mutation burden and RTK–RAS pathway alterations, indicating a more extensive genomic shift with FOLFOX exposure. Direct ancestry comparisons in EOCRC revealed that ancestry-related differences in RTK–RAS alterations were most pronounced in untreated disease, with enrichment of *ERBB2*, *MAPK3*, and *NF1* mutations among untreated Hispanic/Latino patients, while such differences were largely attenuated following FOLFOX treatment. Collectively, these findings highlight strong age-, ancestry-, and treatment-specific modulation of RTK–RAS alterations, underscoring the importance of integrated stratification when evaluating genomic biomarkers in CRC.

### 3.3. RTK–RAS Pathway Alterations by Age at Onset, Ancestry, and FOLFOX Status

The distribution of RTK–RAS pathway alterations was evaluated across H/L and NHW CRC cohorts, stratified by age at diagnosis and exposure to FOLFOX chemotherapy (Table 3a–d).

Within the H/L cohort, RTK–RAS pathway alteration frequencies were high and did not differ significantly by treatment status in either age group. Among EOCRC H/L patients, alterations were detected in 64.4% of FOLFOX-treated cases and 67.3% of non-FOLFOX-treated cases (*p* = 0.8822). Similarly, in LOCRC H/L patients, alteration prevalence was nearly identical between FOLFOX-treated (76.9%) and untreated patients (76.0%; *p* = 1.0), indicating no measurable treatment-associated difference in pathway-level alteration frequency within this ancestry group.

In the NHW cohort, RTK–RAS alterations were also common across all strata. Among EOCRC NHW patients, pathway alterations were observed in 66.4% of FOLFOX-treated cases and 61.3% of non-treated cases (*p* = 0.1916). In LOCRC NHW patients, alteration prevalence was comparable between FOLFOX-treated (69.5%) and non-treated groups (70.3%; *p* = 0.7892), demonstrating stability of pathway-level frequencies regardless of treatment exposure.

Between-ancestry comparisons in EOCRC disease showed no significant differences in RTK–RAS alteration prevalence. Among FOLFOX-treated EOCRC patients, alterations were present in 64.4% of H/L cases and 66.4% of NHW cases (*p* = 0.8432). In the non-FOLFOX-treated EOCRC group, alteration frequencies were similarly comparable between H/L (67.3%) and NHW (61.3%) patients (*p* = 0.4991).

Ancestry-based comparisons in LOCRC disease also revealed no statistically significant differences. Among FOLFOX-treated LOCRC patients, RTK–RAS alterations were detected in 76.9% of H/L cases and 69.5% of NHW cases (*p* = 0.1769). In non-FOLFOX-treated LOCRC patients, alteration prevalence was 76.0% in H/L patients and 70.3% in NHW patients (*p* = 0.4879).

Across all age, ancestry, and treatment strata, the majority of CRC patients harbored at least one RTK–RAS pathway alteration. Although overall pathway-level frequencies were largely conserved, these findings provide an essential framework for interpreting the gene-level, ancestry-specific, and treatment-dependent differences observed in subsequent analyses, underscoring the importance of dissecting RTK–RAS signaling beyond aggregate pathway prevalence.

Across all age-at-onset, ancestry, and treatment-defined subgroups, RTK–RAS pathway alterations were highly prevalent and showed no significant differences in overall frequency by FOLFOX exposure or ancestry. Both Hispanic/Latino and non-Hispanic White patients exhibited stable pathway-level alteration rates in early- and late-onset disease, indicating that FOLFOX treatment and ancestry do not substantially influence aggregate RTK–RAS alteration prevalence. Importantly, this stability at the pathway level contrasts with the pronounced gene-level, ancestry-specific, and treatment-dependent differences observed elsewhere, highlighting the necessity of moving beyond overall pathway frequencies to uncover clinically meaningful heterogeneity within RTK–RAS signaling.

### 3.4. Gene-Level RTK–RAS Alteration Patterns Across Ancestry, Age of Onset, and FOLFOX Exposure

Gene-specific comparisons across RTK–RAS pathway members demonstrated that most loci exhibited broadly similar mutation frequencies across strata, with only a limited subset showing statistically significant treatment- or subgroup-associated differences (Tables S1–S12).

#### 3.4.1. EOCRC H/L: FOLFOX-Treated vs. Not Treated

Within EOCRC H/L patients, two receptor tyrosine kinase/RAS-regulator genes differed by treatment exposure (Table S1). *ERBB2* alterations were less common in FOLFOX-treated EOCRC H/L cases (2.7%) than in untreated EOCRC H/L cases (15.4%; *p* = 0.016). Similarly, *NF1* mutations were reduced in treated EO H/L patients (4.1%) relative to untreated EOCRC H/L patients (19.2%; *p* = 0.014). A second *RTK* gene signal was also observed: *FGFR2* alterations were absent in treated EOCRC H/L patients (0.0%) but present in 7.7% of untreated EOCRC H/L cases (*p* = 0.028). Most other RTK–RAS genes, including *KRAS* (41.1% vs. 34.6%), *NRAS*, *BRAF*, and downstream *MAPK* components, did not differ significantly by FOLFOX exposure.

#### 3.4.2. LOCRC H/L: FOLFOX-Treated vs. Not Treated

In LOCRC H/L patients, gene-level frequencies were largely stable across treatment groups (Table S2). The most notable treatment-associated difference involved *NTRK2*, which was absent in FOLFOX-treated LOCRC H/L cases (0.0%) but detected in 6.0% of untreated LOCRC H/L cases (*p* = 0.043). Other genes, including *KRAS* (~41–43%) and *BRAF* (16.5% vs. 16.0%), were comparable between treated and untreated groups.

#### 3.4.3. Age-of-Onset Contrasts Within H/L

When restricting to FOLFOX-treated H/L patients (EOCRC vs. LOCRC; Table S3), *BRAF* mutations were significantly more frequent in LOCRC disease (16.5%) than EOCRC disease (4.1%; *p* = 0.012), while most other genes were similar. In the non-FOLFOX H/L stratum (EOCRC vs. LOCRC; Table S4), *NF1* alterations were enriched in EOCRC relative to LOCRC (19.2% vs. 4.0%; *p* = 0.028), consistent with the EOCRC-specific *NF1* signal seen in treatment comparisons.

#### 3.4.4. NHW Patterns by Age and Treatment

Across NHW cohorts, most RTK–RAS genes showed modest or no treatment-associated shifts, but EOCRC (Table S5) and LOCRC (Table S6) disease demonstrated several significant differences when comparing treated vs. untreated cases. Specifically in LOCRC, *ERBB3* (3.8% vs. 7.2%; *p* = 0.004), *KIT* (1.5% vs. 4.0%; *p* = 0.004), *RET* (2.1% vs. 5.1%; *p* = 0.002), *ALK* (3.2% vs. 6.0%; *p* = 0.010), *ARAF* (1.3% vs. 2.9%; *p* = 0.038), and *RAF1* (0.9% vs. 2.9%; *p* = 0.004) were all less frequent among LOCRC NHW patients receiving FOLFOX than among those not treated with FOLFOX. Additional LOCRC NHW differences included higher *MAPK1* (0.1% vs. 0.9%; *p* = 0.023) and lower *SOS1* (0.9% vs. 2.3%; *p* = 0.035) in treated vs. untreated patients. In contrast, EOCRC NHW comparisons were more limited, with BRAF higher in LOCRC vs. EOCRC among FOLFOX-treated patients (11.1% vs. 7.2%; *p* = 0.043; Table S7) and similarly higher in LO vs. EO among untreated patients (14.2% vs. 7.9%; *p* = 0.008; Table S8).

#### 3.4.5. Ancestry Contrasts Within EOCRC and LOCRC Strata

Across EOCRC FOLFOX-treated patients (H/L vs. NHW; Table S9), no single RTK–RAS gene differed significantly by ancestry. However, among EOCRC patients not treated with FOLFOX (Table S10), multiple alterations were more frequent in H/L than NHW, including *ERBB2* (15.4% vs. 5.3%; *p* = 0.018), *MAPK3* (5.8% vs. 1.0%; *p* = 0.043), *CBL* (9.6% vs. 1.3%; *p* = 0.0045), and *NF1* (19.2% vs. 6.0%; *p* = 0.0027). In LOCRC comparisons, ancestry-associated differences were limited: in FOLFOX-treated LOCRC patients, MET was more frequent in H/L than NHW (4.4% vs. 1.3%; *p* = 0.048; Table S11), whereas in LOCRC untreated patients, *NTRK2* was higher in H/L than NHW (6.0% vs. 1.4%; *p* = 0.047; Table S12).

These gene-level analyses indicate that while canonical drivers such as *KRAS* and *BRAF* remain prevalent across subgroups, the most prominent subgroup-specific signals involved *ERBB2* and *NF1* in EOCRC H/L disease (particularly among untreated patients), and multiple RTK/RAS-associated loci in LOCRC NHW patients showing reduced mutation frequencies in the FOLFOX-treated stratum.

### 3.5. Mutational Landscape of the RTK–RAS Pathway

#### 3.5.1. EOCRC H/L CRC

**Figure 1:**
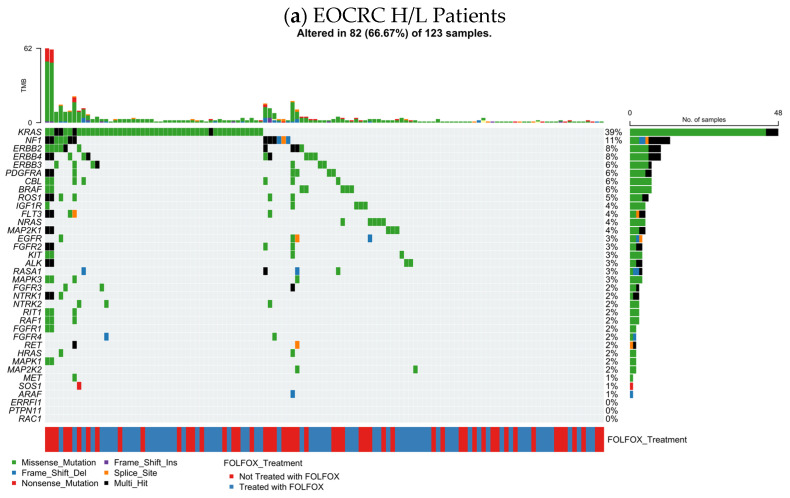

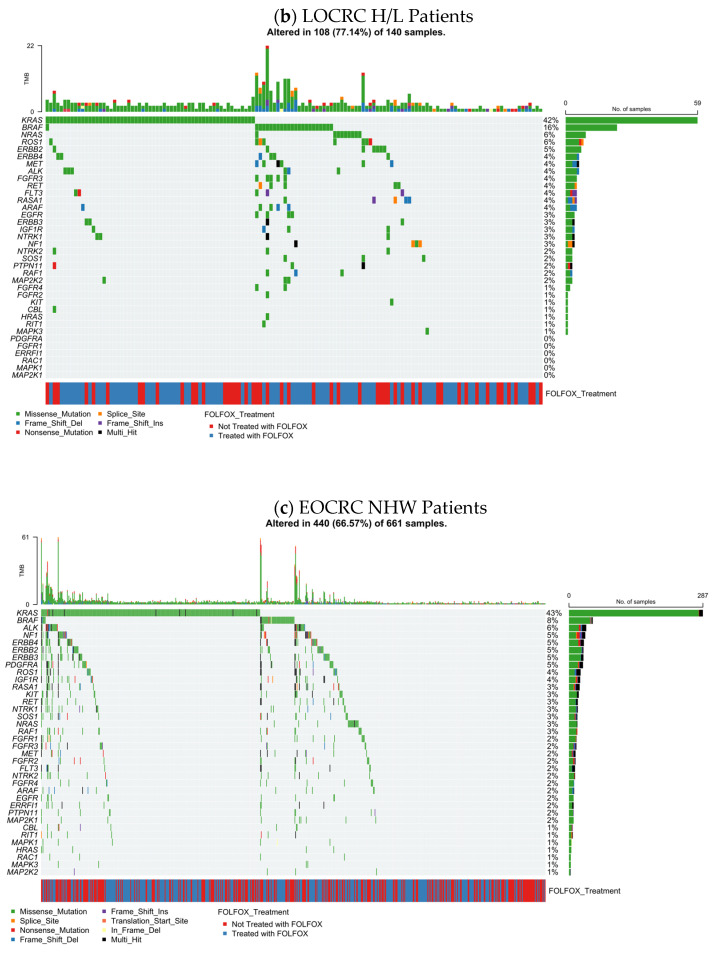

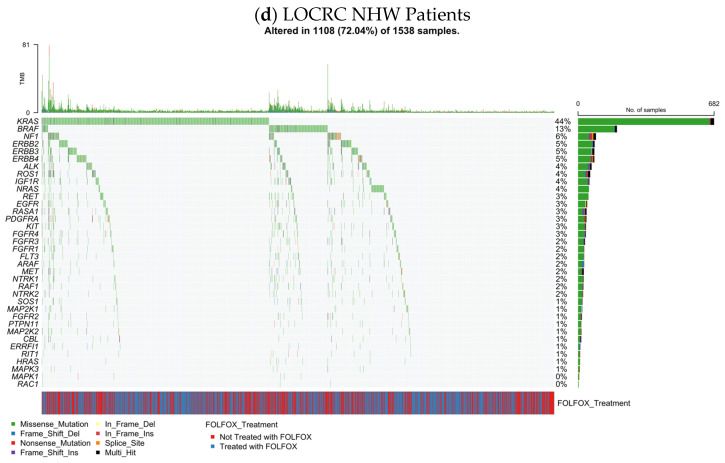
RTK–RAS pathway mutational architecture in Hispanic/Latino colorectal cancer by age at onset. Oncoprint visualizations depict somatic alterations across core RTK–RAS pathway genes in Hispanic/Latino (H/L) colorectal cancer, stratified by early-onset (EOCRC) (**a**,**c**) and late-onset (LOCRC) (**b**,**d**) disease. Each column represents an individual tumor, with rows corresponding to pathway genes and color-coded annotations indicating mutation classes. Upper bar plots summarize tumor mutational burden (TMB), while lower annotations denote FOLFOX treatment exposure. Side panels report gene-level alteration frequencies, enabling comparison of pathway disruption patterns and mutation spectra between EOCRC and LOCRC H/L CRC and Non-Hispanic Whites (NHW).

Figure 1a summarizes the RTK–RAS pathway alteration profile in EOCRC Hispanic/Latino (H/L) CRC (n = 123), integrating per-sample TMB, mutation class, and FOLFOX exposure. Overall, 82 tumors (66.7%) harbored at least one RTK–RAS pathway alteration, indicating that pathway disruption is common in this subgroup.

At the gene level, *KRAS* was the dominant event (39%), largely comprising missense substitutions, consistent with canonical activating variants. A second tier of recurrently altered genes included NF1 (11%) and multiple receptor tyrosine kinases, led by *ERBB2* (8%), *ERBB4* (8%), and *ERBB3* (6%), alongside *PDGFRA* (6%), *CBL* (6%), *BRAF* (6%), and *ROS1* (6%). Additional alterations were observed at lower frequencies across the pathway (generally ~2–4%), including *NRAS*, *EGFR*, *FGFR*-family members, *KIT*, *ALK*, *RET*, *RAF1*, *MAP2K1*, and *MAPK3*, reflecting a broad but shallow tail of RTK–RAS gene involvement.

Across the oncoprint, alterations were predominantly missense, with intermittent truncating (frameshift/nonsense) and multi-hit patterns in select genes (Table S13). TMB values were mostly in the low-to-moderate range, with a small number of higher-TMB outliers that did not cluster within a single driver gene. Importantly, FOLFOX-treated and untreated cases were interspersed throughout the alteration spectrum, without an obvious segregation by treatment status, suggesting that the overall RTK–RAS mutational architecture in EOCRC H/L CRC is not driven by a single FOLFOX-associated genomic signature at the level of this visualization.

#### 3.5.2. LOCRC H/L CRC

The distribution of RTK–RAS pathway alterations in late-onset Hispanic/Latino colorectal cancer (LOCRC; n = 140) is shown (Figure 1b), combining gene-level mutation frequencies with mutation classes, TMB, and FOLFOX treatment annotation. Overall, 108 tumors (77.1%) harbored at least one RTK–RAS pathway alteration, indicating a higher prevalence of pathway disruption in LOCRC compared with EOCRC H/L disease.

Consistent with canonical CRC biology, *KRAS* was the most frequently altered gene (42%), predominantly through missense mutations, reflecting activating hotspot variants. Additional recurrent alterations were observed in *BRAF* (16%) and *NRAS* (6%), together highlighting MAPK-axis engagement in a substantial fraction of tumors. Among receptor tyrosine kinases, *ROS1* (6%), *ERBB2* (5%), *ERBB4* (4%), *ALK* (4%), *FGFR3* (4%), *RET* (4%), and *IGF1R* (3%) contributed to pathway heterogeneity, while downstream regulators such as *NF1* (3%), *RAF1* (3%), and *MAP2K1* (2%) were detected at lower frequencies.

The majority of alterations across genes consisted of missense substitutions (Table S13), with sporadic frameshift, nonsense, splice-site, and multi-hit events, particularly among RTK family members and negative regulators. TMB values were generally low to moderate across samples, with a limited number of higher-TMB outliers that did not cluster around specific RTK–RAS genes.

Assessment of treatment annotation revealed that FOLFOX-treated and untreated tumors were distributed across the full spectrum of RTK–RAS alterations, without clear segregation by gene or mutation class. This pattern suggests that, at the level of pathway-wide mutational architecture, LOCRC H/L CRC exhibits substantial RTK–RAS dysregulation that is not dominated by a single treatment-associated genomic signature, supporting subsequent analyses focused on gene-specific and survival-related effects.

#### 3.5.3. EOCRC NHW CRC

The RTK–RAS mutational landscape in early-onset non-Hispanic White (NHW) colorectal cancer (n = 661) is illustrated (Figure 1c), displaying gene-specific alteration frequencies together with mutation types, TMB, and FOLFOX treatment status. Overall, 440 tumors (66.6%) harbored at least one RTK–RAS pathway alteration, demonstrating that RTK–RAS dysregulation is a dominant molecular feature of EOCRC in NHW patients.

As expected, *KRAS* was the most frequently altered gene (43%), largely through missense mutations, consistent with activating hotspot variants. Additional recurrent alterations were observed in *BRAF* (8%), *ALK* (6%), *NF1* (5%), *ERBB4* (5%), and *ERBB2* (5%), followed by *ERBB3* (5%), *PDGFRA* (5%), and *ROS1* (4%). A long tail of less frequent events involved *IGF1R, RASA1, KIT, RET, NTRK1, SOS1, NRAS, RAF1*, FGFR-family members, *MET*, and downstream MAPK components, each occurring in approximately 1–3% of cases.

Across the cohort, alterations were predominantly missense substitutions (Table S13), with occasional frameshift, nonsense, splice-site, translation start site, and multi-hit events scattered among RTK and RAS-regulatory genes. The TMB distribution was largely low to moderate, with a subset of tumors exhibiting elevated TMB that did not map to a single dominant RTK–RAS gene, suggesting heterogeneous mutational processes rather than pathway-specific hypermutation.

Evaluation of treatment annotation showed that FOLFOX-treated and untreated EOCRC NHW tumors were broadly intermingled across the oncoprint, without clear segregation by gene or mutation class. When considered alongside the EOCRC H/L cohort, EOCRC NHW patients demonstrated a similarly high overall prevalence of RTK–RAS alterations but with differences in the relative contribution of specific receptor tyrosine kinases and regulatory genes, underscoring ancestry-specific variation within a shared RTK–RAS-driven oncogenic framework.

#### 3.5.4. LOCRC NHW CRC

Figure 1d depicts the RTK–RAS alteration profile in LOCRC NHW colorectal cancer (LOCRC; n = 1538), integrating mutation class, TMB, and FOLFOX treatment annotation. Overall, 1108 tumors (72.0%) harbored at least one RTK–RAS pathway alteration, underscoring the pervasive involvement of this signaling axis in LOCRC NHW disease.

Across the cohort, *KRAS* was the most frequently altered gene (44%), overwhelmingly driven by missense substitutions consistent with activating oncogenic variants. Additional recurrent alterations included *BRAF* (13%), NF1 (6%), and several receptor tyrosine kinases, most notably *ERBB2* (5%), *ERBB3* (5%), *ERBB4* (5%), *ROS1* (5%), and *IGF1R* (4%). A broad distribution of lower-frequency events (approximately 1–3%) involved *NRAS, RET, KIT,* FGFR-family members, *ALK, MET, ARAF, RAF1*, and downstream MAPK regulators, reflecting extensive pathway heterogeneity.

The majority of observed variants were missense (Table S13), with smaller contributions from in-frame insertions/deletions, frameshift events, nonsense mutations, splice-site alterations, and multi-hit configurations, particularly among RTK family members and pathway regulators. TMB values were predominantly low to moderate, with a limited number of tumors showing elevated TMB that did not cluster within specific RTK–RAS genes, suggesting diverse mutational processes rather than a single hypermutated pathway-driven subset.

Assessment of treatment status revealed that FOLFOX-treated and untreated LOCRC NHW tumors were broadly interspersed across genes and mutation classes, with no clear segregation by treatment exposure in the overall RTK–RAS mutational landscape. When considered alongside EOCRC NHW CRC, LOCRC disease exhibited a modestly higher prevalence of pathway alterations and a greater contribution from BRAF and receptor tyrosine kinase alterations, highlighting age-associated shifts within an otherwise conserved RTK–RAS-driven oncogenic framework.

### 3.6. Survival Impact of RTK–RAS Pathway Alterations Across Age, Ancestry, and Treatment Groups

We evaluated the association between RTK–RAS pathway alteration status and overall survival (OS) in EOCRC and LOCRC using Kaplan–Meier analysis, stratifying patients by age at onset, ancestry, and exposure to FOLFOX chemotherapy.

**Figure 2:**
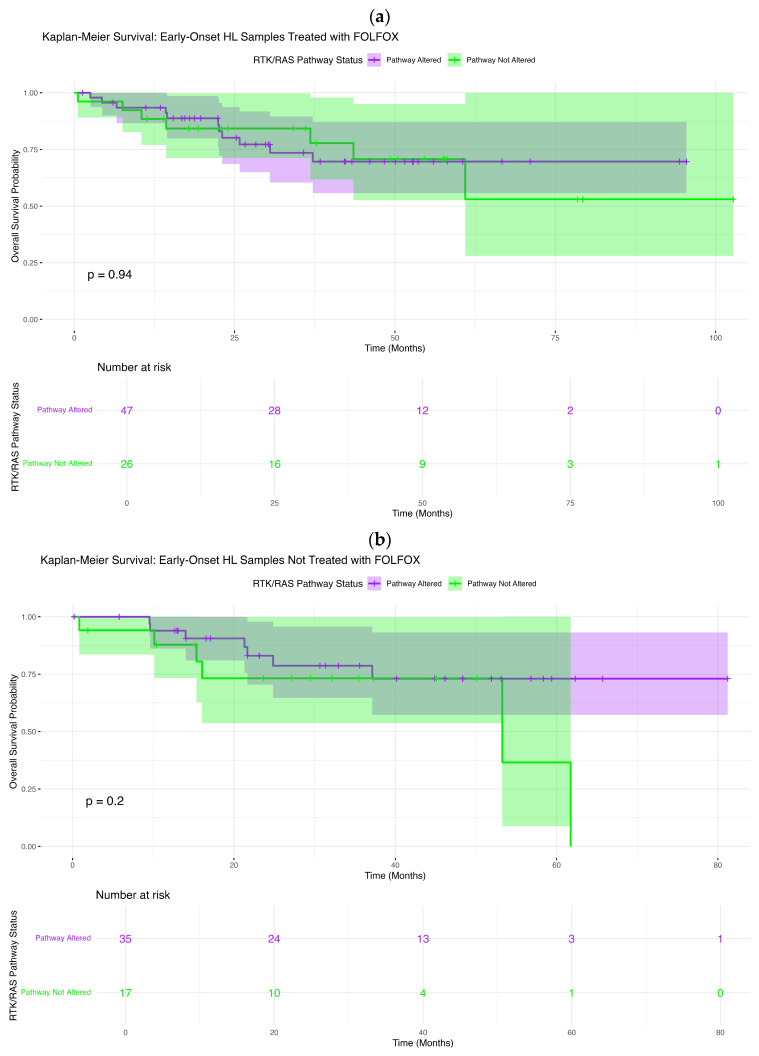

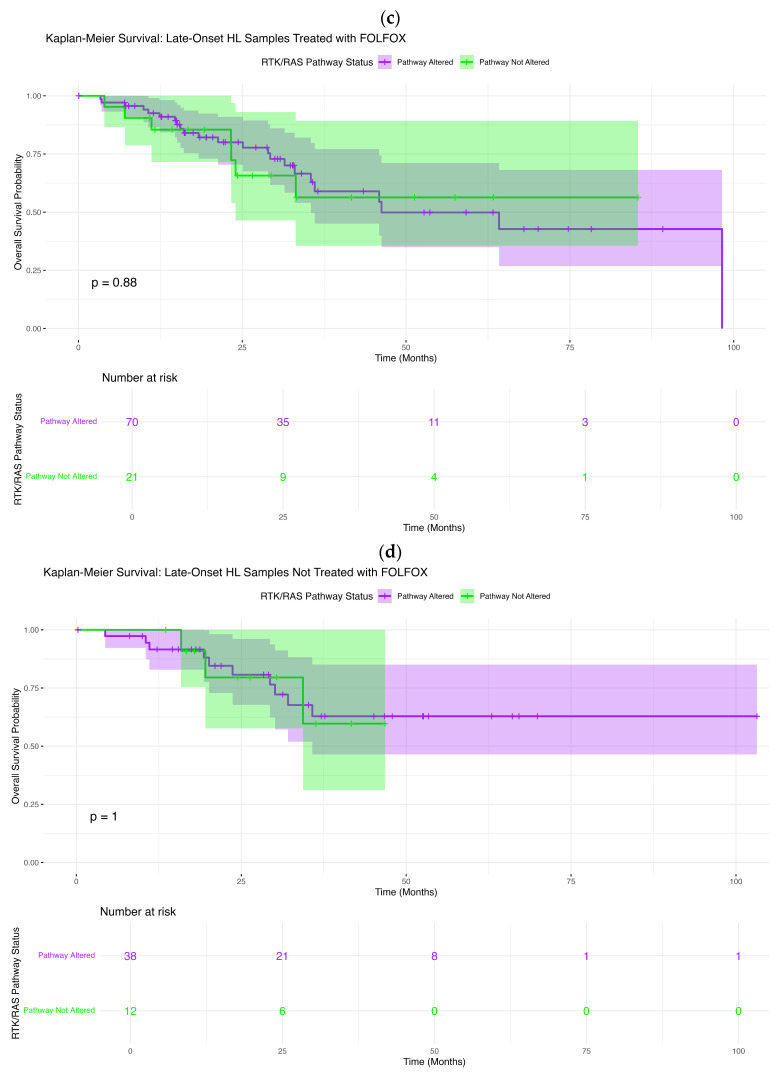

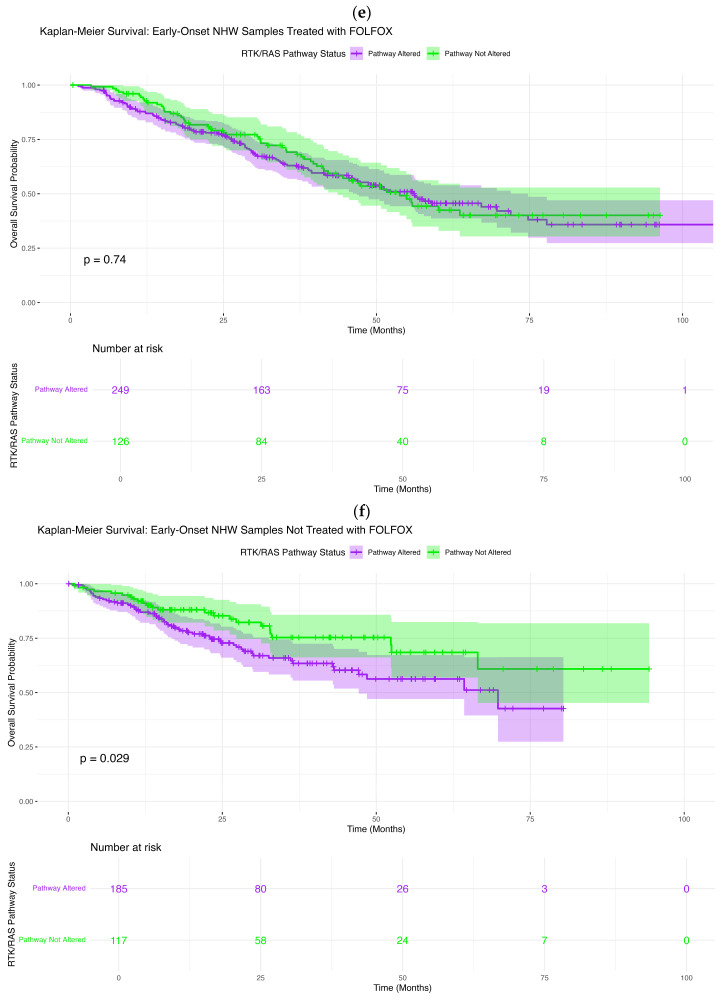

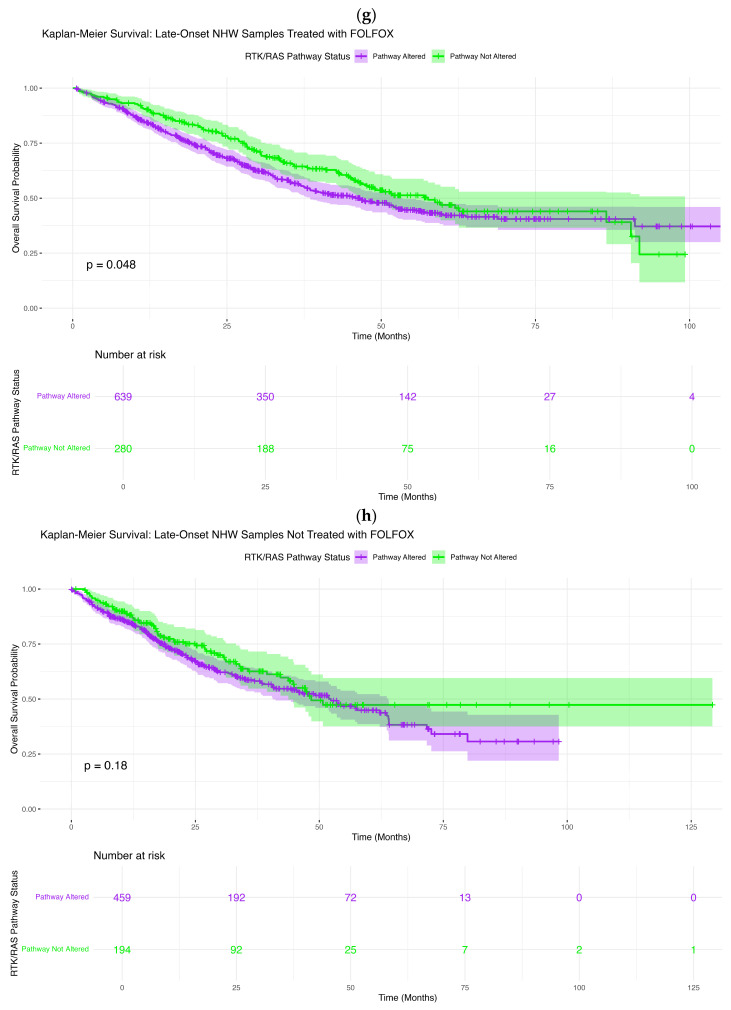
Prognostic significance of RTK–RAS pathway alterations across colorectal cancer subgroups stratified by age at onset, ancestry, and FOLFOX exposure. Kaplan–Meier overall survival curves are presented according to RTK–RAS pathway alteration status (altered vs. non-altered) within clinically and demographically defined CRC subgroups. Panels include: (**a**,**b**) early-onset (EOCRC) Hispanic/Latino (H/L) patients treated with or not treated with FOLFOX; (**c**,**d**) late-onset (LOCRC) H/L patients treated with or not treated with FOLFOX; (**e**,**f**) EOCRC non-Hispanic White (NHW) patients treated with or not treated with FOLFOX; and (**g**,**h**) LOCRC NHW patients treated with or not treated with FOLFOX. Shaded regions denote 95% confidence intervals, and numbers at risk are displayed below each plot to facilitate comparison of subgroup-specific survival trajectories associated with RTK–RAS pathway alterations.

Among EOCRC H/L patients treated with FOLFOX, RTK–RAS pathway alterations were not associated with differences in overall survival (Figure 2a). Survival curves for altered and non-altered tumors were nearly superimposable across the entire follow-up period, with no sustained divergence observed (log-rank *p* = 0.94). Survival probabilities declined gradually and in parallel for both groups, and the wide, overlapping confidence intervals, particularly at later time points, reflected diminishing numbers at risk rather than a biologically meaningful separation. These findings indicate that RTK–RAS alteration status does not influence OS in FOLFOX-treated early-onset H/L patients.

Similarly, in EOCRC H/L patients who did not receive FOLFOX, overall survival did not differ by RTK–RAS pathway status (Figure 2b). Kaplan–Meier curves remained largely overlapping throughout follow-up, and no statistically significant association was detected (log-rank *p* = 0.20). Although a modest separation emerged at later time points, this pattern was driven by a small number of events and was accompanied by broad confidence intervals, limiting interpretability. Collectively, these results suggest that RTK–RAS alterations do not materially affect survival outcomes in early-onset H/L CRC in the absence of oxaliplatin-based chemotherapy.

In LOCRC H/L patients treated with FOLFOX, survival outcomes were likewise independent of RTK–RAS pathway status (Figure 2c). Kaplan–Meier estimates for altered and non-altered tumors closely tracked one another across follow-up, with no significant difference observed (log-rank *p* = 0.88). Minor early fluctuations between curves were not sustained and were accompanied by overlapping confidence intervals, indicating no consistent prognostic effect.

A comparable pattern was observed in LOCRC H/L patients not treated with FOLFOX (Figure 2d). Survival curves for altered and non-altered tumors were virtually indistinguishable across the full duration of follow-up, and statistical testing confirmed the absence of any survival difference (log-rank *p* = 1.0). These findings collectively demonstrate that, across all age and treatment strata within the H/L population, RTK–RAS pathway alteration status does not confer prognostic significance for overall survival.

In contrast, ancestry- and treatment-specific effects emerged within the Non-Hispanic White (NHW) cohort. Among early-onset NHW CRC patients treated with FOLFOX, RTK–RAS pathway alterations were not associated with OS differences (Figure 2e). The Kaplan–Meier curves followed closely aligned trajectories, and formal comparison showed no significant association (log-rank *p* = 0.74). Minor divergence at intermediate time points was accompanied by extensive overlap of confidence intervals, suggesting that these fluctuations do not reflect a robust biological effect.

However, among EOCRC NHW patients who did not receive FOLFOX, RTK–RAS pathway alteration status was significantly associated with inferior overall survival (Figure 2f). Patients with RTK–RAS-altered tumors exhibited a clear and persistent survival disadvantage compared with non-altered patients, as evidenced by early separation of Kaplan–Meier curves that widened over time (log-rank *p* = 0.029). Although confidence intervals broadened at later follow-up due to decreasing numbers at risk, the sustained divergence supports a negative prognostic impact of RTK–RAS alterations in untreated early-onset NHW CRC.

Conversely, in LOCRC NHW patients treated with FOLFOX, RTK–RAS pathway alterations were associated with improved overall survival (Figure 2g). Altered tumors demonstrated consistently higher survival probabilities compared with non-altered tumors, with progressive separation of Kaplan–Meier curves over follow-up (log-rank *p* = 0.048). While confidence intervals widened toward the tail of follow-up, the sustained and directional curve separation suggests a favorable prognostic association of RTK–RAS alterations in this treatment-defined subgroup, contrasting sharply with the adverse effect observed in untreated early-onset NHW disease.

Finally, among LOCRC NHW patients who did not receive FOLFOX, RTK–RAS pathway status was not significantly associated with OS (Figure 2h). Survival curves for altered and non-altered tumors followed largely parallel trajectories, with only modest late separation and substantial overlap of confidence intervals (log-rank *p* = 0.18). Although non-altered patients appeared to maintain slightly higher long-term survival, this trend did not reach statistical significance, indicating no clear prognostic effect in this subgroup.

These analyses reveal a striking age-, ancestry-, and treatment-dependent relationship between RTK–RAS pathway alterations and survival. RTK–RAS alterations conferred adverse prognosis in untreated early-onset NHW CRC, were associated with improved survival in FOLFOX-treated late-onset NHW CRC, and showed no measurable survival impact across all Hispanic/Latino subgroups, regardless of age or treatment exposure. These findings highlight the context-dependent nature of RTK–RAS pathway biology and underscore the importance of integrating ancestry, age at onset, and chemotherapy exposure when interpreting genomic prognostic biomarkers in CRC.

### 3.7. AI-Driven Exploratory Interrogation and Hypothesis Refinement

To enable efficient hypothesis generation prior to formal statistical testing, the AI-HOPE [38] and AI-HOPE–RTK–RAS [39] platforms were applied to conduct an automated, post hoc exploration of the integrated CRC dataset. Through natural language-based querying of harmonized clinical, molecular, and treatment-related variables, the AI framework rapidly surfaced candidate associations with potential biological and clinical relevance, which were subsequently evaluated using confirmatory statistical methods.

As an initial finding, AI-guided interrogation identified a possible survival disadvantage associated with RTK–RAS pathway alterations in EOCRC NHW CRC patients who had not received FOLFOX chemotherapy. To formally assess this observation, a targeted case–control analysis was performed, defining cases as EOCRC NHW patients harboring at least one RTK–RAS pathway alteration (n = 185) and controls as EOCRC NHW patients without such alterations (n = 117), all without FOLFOX exposure. Kaplan–Meier survival analysis demonstrated significantly inferior overall survival among patients with RTK–RAS-altered tumors compared with their non-altered counterparts (log-rank *p* = 0.0288) (Figure S2). Notably, survival curves diverged early in follow-up, with altered cases experiencing a more rapid decline in survival probability, supporting a potential prognostic role for RTK–RAS pathway dysregulation independent of oxaliplatin-based treatment.

Next, the AI platform executed a structured evaluation of cohort composition under tightly defined clinical and genomic constraints to determine whether preliminary pathway enrichment signals persisted across EOCRC subgroups. This analysis revealed that, for both case and control cohorts, samples satisfying the analytic context constituted a small and highly specific subset of the overall dataset, underscoring the precision of AI-guided cohort selection and minimal inclusion of out-of-context samples. Subsequent Fisher’s exact testing did not demonstrate significant enrichment of the queried genomic feature between the compared groups (Figure S3). These findings illustrate how AI-facilitated exploratory signals can be rigorously examined through transparent cohort construction and pre-statistical validation, enabling discrimination between true biological patterns and chance findings.

Concurrently, AI-driven global screening revealed a wide range of clinical and molecular features that differed significantly between early-onset Hispanic/Latino and early-onset non-Hispanic White colorectal cancer patients, independent of treatment exposure (*p* < 0.05). These differences encompassed demographic variables (ethnicity and race), diagnostic and tumor characteristics (cancer type, diagnostic classification, sample type, and stage at presentation), as well as clinical outcomes (overall survival status and event occurrence). In addition, several key driver genes—including *SMAD4, APC, TP53, KRAS*, and *BRAF*—showed ancestry-specific differences in mutation frequency (Figure S4).

Tumor anatomical site and stage at diagnosis emerged as particularly prominent distinguishing factors, aligning with prior reports of ancestry-associated variation in EOCRC CRC presentation. Together, these results highlight the capacity of AI-guided interrogation to uncover multidimensional heterogeneity across populations and to systematically prioritize variables for downstream analyses.

Finally, AI-driven screening highlighted a potential ancestry-related difference in BRAF mutation frequency among EOCRC patients treated with FOLFOX. This observation prompted a focused case–control comparison between EOCRC H/L patients (n = 73) and EOCRC NHW patients (n = 375) who received FOLFOX chemotherapy. Statistical evaluation using Fisher’s exact test revealed a differential distribution of *BRAF* mutations between the two ancestry groups (Figure S5). Although *BRAF* alterations were infrequent overall, the observed imbalance suggests possible ancestry-associated differences in RTK–RAS pathway architecture within this clinically relevant treatment subgroup, meriting further biological and therapeutic investigation.

In summary, these AI-derived exploratory analyses informed the selection of clinically meaningful subgroup comparisons and guided the prioritization of variables for formal statistical testing. By automating cohort definition, filtering, and visualization across complex clinical and genomic dimensions, the AI-HOPE [38] framework reduced manual analytic burden, improved reproducibility, and streamlined the progression from hypothesis generation to confirmatory precision oncology analysis.

## 4. Discussion

In this study, we applied an AI-enabled precision oncology workflow, AI-HOPE [38] and the pathway-specialized AI-HOPE–RTK–RAS agent [39], to interrogate RTK–RAS alterations in colorectal cancer (CRC) at the intersection of age at onset, ancestry, and FOLFOX exposure. By integrating harmonized clinical annotations with pathway-level somatic mutation calls across 2515 cases, we identified context-dependent genomic patterns and divergent survival associations that would be difficult to resolve without high-dimensional subgrouping. Collectively, our results support a model in which RTK–RAS dysregulation is common across CRC, yet its clinical meaning varies substantially depending on treatment exposure and patient subgroup.

### 4.1. RTK–RAS Alterations Show Treatment- and Age-Dependent Prognostic Polarity in NHW CRC

A central observation was that RTK–RAS pathway status was not uniformly prognostic across the cohort. Instead, survival associations emerged only in specific strata. In EOCRC NHW patients not treated with FOLFOX, RTK–RAS alterations were associated with inferior overall survival (log-rank *p* ≈ 0.029). This suggests that, in the absence of oxaliplatin-based therapy, RTK–RAS pathway disruption may act as a surrogate for more aggressive biology, potentially reflecting higher baseline signaling flux through RAS/RAF/MAPK, greater metastatic competence, or enrichment for adverse molecular co-alteration profiles that were not explicitly modeled here.

In contrast, in LOCRC NHW patients treated with FOLFOX, RTK–RAS alterations were associated with improved overall survival (*p* ≈ 0.048), indicating an opposite directional effect. One biologically plausible explanation is that, in LO disease, certain RTK–RAS-altered tumors may remain more chemosensitive or may represent a subgroup with distinct clinical trajectories (e.g., differential metastatic patterns, treatment sequencing, or tumor burden at therapy initiation). Alternatively, RTK–RAS alteration status may track with a broader molecular state that interacts favorably with fluoropyrimidine/oxaliplatin exposure in older NHW patients. Regardless of mechanism, this polarity highlights an important principle: pathway alteration status can be prognostic in one context and neutral, or even favorable, in another, reinforcing the need for subgroup-aware biomarker interpretation.

### 4.2. Gene-Level Differences Suggest Selection Pressures Linked to FOLFOX Exposure

Although overall RTK–RAS pathway alteration prevalence was high across strata, gene-level analyses revealed selective patterns consistent with treatment-associated pressures or confounding by treatment selection. In EOCRC H/L, *ERBB2* and *NF1* alterations were less frequent among FOLFOX-treated versus untreated patients, and in LO H/L, *NTRK2* alterations were depleted in FOLFOX-exposed cases. In NHW, the most prominent treatment-associated depletion occurred in LO NHW, where multiple RTK–RAS genes (e.g., *ERBB3, KIT, IGF1R, RET, ALK, FLT3, ERRFI1, ARAF, RAF1*) were less enriched among FOLFOX-treated patients. These consistent reductions across multiple loci suggest that FOLFOX-exposed subgroups may differ systematically from unexposed groups, either because treatment selection correlates with clinical variables (stage, resectability, comorbidities, metastatic site), or because particular molecular contexts are underrepresented among FOLFOX-treated patients due to prior therapy, lineage effects, or survivorship bias. Future analyses incorporating stage-stratified models, regimen timing, and metastatic status will help disentangle true gene-treatment interactions from treatment selection effects.

### 4.3. Ancestry-Associated Patterns Are Most Evident in Untreated EOCRC Disease

Between-ancestry comparisons indicated that ancestry-linked differences were not uniform across therapy strata. Notably, among EOCRC patients not treated with FOLFOX, H/L patients showed enrichment of select RTK–RAS genes (including *ERBB2, MAPK3, CBL*, and *NF1*) relative to NHW. This pattern was less apparent in FOLFOX-treated EOCRC patients, suggesting that chemotherapy exposure (or the clinical scenarios that lead to exposure) may partially obscure or reshape detectable ancestry-linked differences. These observations align with the broader view that ancestry-associated disparities in CRC are likely multi-factorial, reflecting tumor biology, host factors, environmental exposures, and care pathways, such that ancestry signals may be most visible in strata with fewer treatment-related filters.

The divergent prognostic effects of RTK–RAS alterations observed across age and treatment contexts likely reflect fundamental differences in tumor biology and pathway dependency. In untreated early-onset non-Hispanic White colorectal cancer, RTK–RAS alterations may mark tumors with heightened signaling plasticity, increased proliferative capacity, and intrinsic resistance to cellular stress, thereby conferring a more aggressive natural history in the absence of systemic therapy. In contrast, in late-onset disease treated with FOLFOX, RTK–RAS-altered tumors may exhibit enhanced vulnerability to oxaliplatin- and fluoropyrimidine-induced DNA damage, replication stress, and mitotic catastrophe, particularly in the context of age-related changes in DNA repair capacity and tumor microenvironmental interactions. Chemotherapy exposure may therefore function as a selective pressure that either suppresses RTK–RAS-driven oncogenic clones or converts pathway activation into a liability rather than a survival advantage. Together, these findings suggest that the prognostic impact of RTK–RAS alterations is not intrinsic but context-dependent, shaped by age at onset and treatment exposure, and underscore the importance of integrating biological timing and therapeutic context when interpreting pathway-level biomarkers in precision oncology.

### 4.4. Implications for Precision Oncology: RTK–RAS Status May Inform Subgroup-Specific Risk and Therapy Interpretation

From a clinical standpoint, our findings argue against using a single “RTK–RAS altered” label as a universal prognostic biomarker across CRC. Instead, RTK–RAS pathway alterations may serve as subgroup-specific markers that modify risk in a way that depends on age and treatment context. The adverse survival association in untreated EOCRC NHW disease suggests a potential use case for RTK–RAS status in identifying higher-risk patients who might benefit from intensified surveillance or alternative systemic strategies, while the favorable association in FOLFOX-treated LO NHW disease raises the possibility that, in some older patients, RTK–RAS alterations track with tumors that respond better to standard chemotherapy. These hypotheses require validation in independent cohorts and, ideally, in analyses that incorporate additional clinical covariates, lines of therapy, and metastatic setting.

These findings suggest that RTK–RAS alterations should not be interpreted as static biomarkers but rather as context-dependent signals whose relevance varies by age at onset, ancestry, and treatment exposure. In early-onset colorectal cancer, particularly among untreated patients, the presence of RTK–RAS alterations may identify individuals with more aggressive disease biology who could benefit from earlier systemic therapy or intensified surveillance. Conversely, in late-onset disease treated with FOLFOX, RTK–RAS alterations may mark tumors that derive greater benefit from oxaliplatin-based chemotherapy, supporting their potential use as predictive biomarkers in this setting. These results also highlight the importance of expanding biomarker testing beyond single-gene assessments to pathway-level profiling, enabling more refined risk stratification and therapeutic tailoring. Importantly, incorporating age- and ancestry-aware interpretation of RTK–RAS alterations into clinical workflows could help reduce overtreatment in low-risk groups while optimizing treatment selection for populations that have historically been underrepresented in precision oncology studies.

### 4.5. AI-HOPE as an Enabling Framework for Subgroup-Aware Biomarker Discovery

A key contribution of this work is methodological: the AI-HOPE [38]/AI-HOPE–RTK–RAS [39] agents accelerated cohort construction, stratified querying, and rapid testing of clinically meaningful hypotheses across numerous intersections of ancestry, age, and treatment. Importantly, the AI-driven workflow did not replace statistical inference; rather, it functioned as a scalable discovery layer that identifies candidate signals, constructs transparent case–control subsets, and prioritizes comparisons for confirmatory testing. This approach is particularly relevant for EOCRC and health-equity research, where meaningful subgrouping can quickly lead to sparse strata and complex multi-parameter definitions that are cumbersome to operationalize manually.

### 4.6. Limitations and Future Directions

Several limitations should be considered. First, although the overall cohort was large, certain subgroup analyses, especially when stratifying by ancestry, age, and treatment, reduced effective sample sizes, which can widen confidence intervals and increase susceptibility to instability. Second, FOLFOX exposure was treated as a binary annotation without granular details (line of therapy, dosing intensity, duration, adjuvant vs. metastatic use, and sequencing with targeted agents), limiting causal interpretation of treatment interactions. Third, our RTK–RAS definition relied on protein-altering mutations and did not incorporate other alteration classes (copy-number changes, fusions, or expression-based activation), which may be important for RTK biology. Finally, because these were retrospective, multi-cohort public datasets, residual confounding by clinical covariates and ascertainment differences is unavoidable.

In interpreting ancestry-associated findings, it is important to recognize methodological considerations related to ancestry classification. While self-reported ethnicity and structured annotations were prioritized, surname-based ancestry inference was applied in a limited subset of cases to supplement missing metadata. Although this approach is commonly used in population-level health disparities research, it carries acknowledged limitations, including the potential for misclassification, particularly within small or admixed subgroups.

This study’s findings should be interpreted in the context of its retrospective design and the inherent limitations of non-randomized treatment assignment. FOLFOX exposure was determined by real-world clinical decision-making and may reflect underlying differences in disease stage, comorbid conditions, performance status, treatment era, and physician or patient preferences. As a result, residual confounding related to treatment selection cannot be fully excluded, even with stratification by age at onset, ancestry, and key clinical variables. These factors may influence both the likelihood of receiving chemotherapy and observed outcomes, potentially biasing estimates of prognostic or predictive effects. Accordingly, the associations reported here should be viewed as hypothesis-generating and warrant prospective validation in well-annotated cohorts or clinical trials specifically designed to evaluate age-, ancestry-, and treatment-specific biomarker effects.

Future work should (i) validate these subgroup-specific survival associations in independent ancestrally diverse cohorts; (ii) incorporate richer treatment timelines and metastatic context; (iii) extend RTK–RAS alteration calls to include CNAs/fusions where available; and (iv) integrate microenvironmental and functional context using emerging modalities such as spatial profiling and single-cell analyses to clarify mechanisms linking RTK–RAS dysregulation to chemotherapy response and survival.

## 5. Conclusions

This AI-enabled analysis suggests that the prognostic relevance of RTK–RAS pathway alterations is highly context-dependent, with survival associations that vary according to age at onset, ancestry, and chemotherapy exposure. While these findings highlight the potential importance of interpreting pathway-level biomarkers within well-defined clinical strata, they should be viewed as hypothesis-generating given the retrospective study design. Our results underscore the utility of AI-assisted analytic frameworks for scalable, subgroup-aware biomarker discovery in early-onset colorectal cancer, and they motivate future prospective validation and functional studies to clarify the biological and clinical significance of these observations.

## Figures and Tables

**Table 1:** Baseline Clinical and Demographic Characteristics of the Study Cohorts.

Category	Characteristic	H/L, n = 266	NHW, n = 2249
Demographics	Sex—Male	158 (59.4%)	1267 (56.3%)
	Sex—Female	108 (40.6%)	982 (43.7%)
Tumor Classification	Colon adenocarcinoma	164 (61.7%)	1328 (59.0%)
	Rectal adenocarcinoma	64 (24.1%)	646 (28.7%)
	Colorectal adenocarcinoma (NOS)	38 (14.3%)	275 (12.2%)
Disease Stage at Diagnosis	Stage I–III	156 (58.6%)	1236 (55.0%)
	Stage IV	108 (40.6%)	1005 (44.7%)
	Not available	2 (0.8%)	8 (0.4%)
Microsatellite Status	MSS	200 (75.2%)	1940 (86.3%)
	MSI-H	21 (7.9%)	209 (9.3%)
	Indeterminate	10 (3.8%)	57 (2.5%)
	Not available	35 (13.2%)	43 (1.9%)
Age at Onset & FOLFOX Exposure	EOCRC (<50), FOLFOX-treated	73 (27.4%)	375 (16.7%)
	LOCRC (≥50), FOLFOX-treated	91 (34.2%)	919 (40.9%)
	EOCRC (<50), not FOLFOX-treated	52 (19.5%)	302 (13.4%)
	LOCRC (≥50), not FOLFOX-treated	50 (18.8%)	653 (29.0%)
Sample Characteristics	Primary tumor sequenced	266 (100.0%)	2249 (100.0%)
Ethnicity Annotation	Spanish/Hispanic NOS	230 (86.5%)	0 (0.0%)
	Mexican (incl. Chicano)	30 (11.3%)	0 (0.0%)
	Hispanic or Latino (specified)	2 (0.8%)	0 (0.0%)
	Other Spanish/Hispanic	1 (0.4%)	0 (0.0%)
	Spanish surname only	3 (1.1%)	0 (0.0%)
	Non-Spanish/Non-Hispanic	0 (0.0%)	2249 (100.0%)

**Table 2:** Comparative clinical, genomic, and RTK–RAS pathway features across early (EOCRC) and late (LOCRC) onset colorectal cancer subgroups stratified by ancestry and FOLFOX exposure. This table summarizes key clinical and molecular differences, including median age at diagnosis, mutation burden metrics (total mutation count, tumor mutational burden [TMB], and fraction of genome altered [FGA]), and the prevalence of selected RTK–RAS-related gene alterations, across the following comparisons: (**a**) Early-onset CRC (EOCRC) and LOCRC within Hispanic/Latino (H/L) patients, stratified by FOLFOX treatment status; (**b**) EOCRC and LOCRC within Non-Hispanic White (NHW) patients, stratified by FOLFOX treatment status; (**c**) ancestry-based comparisons between H/L and NHW patients within EOCRC, shown separately for FOLFOX-treated and non-FOLFOX-treated groups.

(**a**)						
**Clinical Feature**	**Early-Onset Hispanic/Latino** **Treated with FOLFOX** **n (%)**	**Early-Onset Hispanic/Latino** **Not Treated with FOLFOX** **n (%)**	***p*** **-Value**	**Late-Onset Hispanic/Latino** **Treated with FOLFOX** **n (%)**	**Late-Onset Hispanic/Latino** **Not Treated with FOLFOX** **n (%)**	***p*** **-Value**
Median Diagnosis Age (IQR)	42 (36–47)	40 (34–43)	0.05411	59 (54–66)	62 (56–70)	0.04865
Median Mutation Count	7 (5–8)	7 (5–20)	0.09735	8 (6–9) [NA = 1]	7 (5.25–9)	0.6507
Median TMB (IQR)	6.3 (4.5–7.8) [NA = 15]	5.5 (3.4–8.3) [NA = 2]	0.1719	6.1 (4.9–7.8) [NA = 10]	6.9 (5.6–9.0) [NA = 2]	0.04389
Median FGA	0.18 (0.03–0.27) [NA = 6]	0.19 (0.03–0.29)	0.7661	0.15 (0.06–0.25) [NA = 7]	0.21 (0.04–0.3) [NA = 2]	0.5464
ERBB2 Mutation						
Present	2 (2.7%)	8 (15.4%)	0.01626	1 (1.1%)	0 (0.0%)	1
Absent	71 (97.3%)	44 (84.6%)		90 (98.9%)	50 (100.0%)	
NF1 Mutation						
Present	3 (4.1%)	10 (19.2%)	0.01437	0 (0.0%)	2 (4.0%)	0.1241
Absent	70 (95.9%)	42 (80.8%)		91 (100.0%)	48 (96.0%)	
NTRK2 Mutation						
Present	1 (1.4%)	2 (3.8%)	0.5699	0 (0.0%)	3 (6.0%)	0.04286
Absent	72 (98.6%)	50 (96.2%)		91 (100.0%)	47 (94.0%)	
(**b**)						
**Clinical Feature**	**Early-Onset NHW** **Treated with FOLFOX** **n (%)**	**Early-Onset NHW** **Not Treated with FOLFOX** **n (%)**	***p*** **-Value**	**Late-Onset NHW** **Treated with FOLFOX** **n (%)**	**Late-Onset NHW** **Not Treated with FOLFOX** **n (%)**	***p*** **-Value**
Median Diagnosis Age (IQR)	43 (37–48)	44 (38–47)	0.5646	63 (57–69)	66 (57–74)	4.146 × 10^−7^
Median Mutation Count	6 (5–8) [NA = 4]	7 (5–9) [NA = 2]	0.1258	7 (5–9) [NA = 10]	8 (6–12) [NA = 3]	1.22 × 10^−5^
Median TMB (IQR)	5.7 (4.1–6.9)	5.7 (4.1–7.8)	0.4214	6.1 (4.3–8.2)	6.6 (4.9–10.4)	0.0002854
Median FGA	0.14 (0.04–0.24) [NA = 4]	0.15 (0.04–0.23) [NA = 2]	0.5589	0.16 (0.06–0.28) [NA = 6]	0.15 (0.05–0.27) [NA = 5]	0.1929
ERBB3 Mutation						
Present	14 (3.7%)	17 (5.6%)	0.3231	35 (3.8%)	47 (7.2%)	0.004198
Absent	361 (96.3%)	285 (94.4%)		884 (96.2%)	606 (92.8%)	
KIT Mutation						
Present	8 (2.1%)	13 (4.3%)	0.04502	14 (1.5%)	26 (4.0%)	0.003883
Absent	367 (97.9%)	289 (95.7%)		905 (98.5%)	627 (96.0%)	
IGF1R Mutation						
Present	8 (2.1%)	16 (5.3%)	0.04502	28 (3.0%)	29 (4.4%)	0.1867
Absent	367 (97.9%)	286 (94.7%)		891 (97.0%)	624 (95.6%)	
RET Mutation						
Present	8 (2.1%)	11 (3.6%)	0.3433	19 (2.1%)	33 (5.1%)	0.001813
Absent	367 (97.9%)	291 (96.4%)		900 (97.9%)	620 (94.9%)	
ALK Mutation						
Present	16 (4.3%)	21 (7.0%)	0.1742	29 (3.2%)	39 (6.0%)	0.009893
Absent	359 (95.7%)	281 (93.0%)		890 (96.8%)	614 (94.0%)	
FLT3 Mutation						
Present	8 (2.1%)	11 (3.6%)	0.3433	19 (2.1%)	33 (5.1%)	0.001813
Absent	367 (97.9%)	291 (96.4%)		900 (97.9%)	620 (94.9%)	
ERRFI1 Mutation						
Present	2 (0.5%)	8 (2.6%)	0.02756	6 (0.7%)	6 (0.9%)	0.7619
Absent	373 (99.5%)	294 (97.4%)		913 (99.3%)	647 (99.1%)	
ARAF Mutation						
Present	7 (1.9%)	4 (1.3%)	0.7623	12 (1.3%)	19 (2.9%)	0.03847
Absent	368 (98.1%)	298 (98.7%)		907 (98.7%)	634 (97.1%)	
RAF1 Mutation						
Present	9 (2.4%)	9 (3.0%)	0.8211	8 (0.9%)	19 (2.9%)	0.004111
Absent	366 (97.6%)	293 (97.0%)		911 (99.1%)	634 (97.1%)	
(**c**)						
**Clinical Feature**	**Early-Onset Hispanic/Latino** **Treated with FOLFOX** **n (%)**	**Early-Onset NHW** **Treated with FOLFOX** **n (%)**	***p*** **-Value**	**Early-Onset Hispanic/Latino** **Not Treated with FOLFOX** **n (%)**	**Early-Onset NHW** **Not Treated with FOLFOX** **n (%)**	***p*** **-Value**
Median Diagnosis Age (IQR)	42 (36–47)	43 (37–48)	0.08467	40 (34–43)	44 (38–47)	0.0006016
Median Mutation Count	7 (5–8)	6 (5–8) [NA = 4]	0.942	7 (5–20)	7 (5–9) [NA = 2]	0.2601
Median TMB (IQR)	6.3 (4.5–7.8) [NA = 15]	5.7 (4.1–6.9)	0.05806	5.5 (3.4–8.3) [NA = 2]	5.7 (4.1–7.8)	0.5732
Median FGA	0.18 (0.03–0.27) [NA = 6]	0.14 (0.04–0.24) [NA = 4]	0.5556	0.19 (0.03–0.29)	0.15 (0.04–0.23) [NA = 2]	0.3612
ERBB2 Mutation						
Present	2 (2.7%)	15 (4.0%)	1	8 (15.4%)	16 (5.3%)	0.01761
Absent	71 (97.3%)	360 (96.0%)		44 (84.6%)	286 (94.7%)	
MAPK3 Mutation						
Present	1 (1.4%)	1 (0.3%)	0.2996	3 (5.8%)	3 (1.0%)	0.04335
Absent	72 (98.6%)	374 (99.7%)		49 (94.2%)	299 (99.0%)	
NF1 Mutation						
Present	3 (4.1%)	17 (4.5%)	1	10 (19.2%)	18 (6.0%)	0.002728
Absent	70 (95.9%)	358 (95.5%)		42 (80.8%)	284 (94.0%)	

**Table 3:** Distribution of RTK–RAS pathway alteration status across colorectal cancer (CRC) subgroups defined by age at diagnosis, ancestry, and FOLFOX exposure. This table presents pathway-level frequencies of RTK–RAS alterations (present vs. absent) among CRC patients stratified by early-onset (EOCRC, <50 years) and LOCRC (≥50 years) disease, Hispanic/Latino (H/L) versus non-Hispanic White (NHW) ancestry, and receipt of FOLFOX chemotherapy. Sub-analyses include: (**a**) treatment-stratified comparisons within early- and LOCRC H/L patients; (**b**) treatment-stratified comparisons within EOCRC and LOCRC NHW patients; (**c**) ancestry-based comparisons among EOCRC patients by FOLFOX treatment status; and (**d**) ancestry-based comparisons among LOCRC patients by FOLFOX treatment status. Reported values reflect the proportion of patients harboring at least one RTK–RAS pathway alteration within each subgroup, with statistical comparisons performed using chi-square or Fisher’s exact tests, as appropriate.

(**a**)						
**Pathway Alterations**	**Early-Onset Hispanic/Latino** **Treated with FOLFOX** **n (%)**	**Early-Onset Hispanic/Latino** **Not Treated with FOLFOX** **n (%)**	***p*** **-Value**	**Late-Onset Hispanic/Latino** **Treated with FOLFOX** **n (%)**	**Late-Onset Hispanic/Latino** **Not Treated with FOLFOX** **n (%)**	***p*** **-Value**
RTK/RAS Alterations Present	47 (64.4%)	35 (67.3%)	0.8822	70 (76.9%)	38 (76.0%)	1
RTK/RAS Alterations Absent	26 (35.6%)	17 (32.7%)		21 (23.1%)	12 (24.0%)	
(**b**)						
**Pathway Alterations**	**Early-Onset NHW** **Treated with FOLFOX** **n (%)**	**Early-Onset NHW** **Not Treated with FOLFOX** **n (%)**	***p*** **-Value**	**Late-Onset NHW** **Treated with FOLFOX** **n (%)**	**Late-Onset NHW** **Not Treated with FOLFOX** **n (%)**	***p*** **-Value**
RTK/RAS Alterations Present	249 (66.4%)	185 (61.3%)	0.1916	639 (69.5%)	459 (70.3%)	0.7892
RTK/RAS Alterations Absent	126 (33.6%)	117 (38.7%)		280 (30.5%)	194 (29.7%)	
(**c**)						
**Pathway Alterations**	**Early-Onset Hispanic/Latino** **Treated with FOLFOX** **n (%)**	**Early-Onset NHW** **Treated with FOLFOX** **n (%)**	***p*** **-Value**	**Early-Onset Hispanic/Latino** **Not Treated with FOLFOX** **n (%)**	**Early-Onset NHW** **Not Treated with FOLFOX** **n (%)**	***p*** **-Value**
RTK/RAS Alterations Present	47 (64.4%)	249 (66.4%)	0.8432	35 (67.3%)	185 (61.3%)	0.4991
RTK/RAS Alterations Absent	26 (35.6%)	126 (33.6%)		17 (32.7%)	117 (38.7%)	
(**d**)						
**Pathway Alterations**	**Late-Onset Hispanic/Latino** **Treated with FOLFOX** **n (%)**	**Late-Onset NHW** **Treated with FOLFOX** **n (%)**	***p*** **-Value**	**Late-Onset Hispanic/Latino** **Not Treated with FOLFOX** **n (%)**	**Late-Onset NHW** **Not Treated with FOLFOX** **n (%)**	***p*** **-Value**
RTK/RAS Alterations Present	70 (76.9%)	639 (69.5%)	0.1769	38 (76.0%)	459 (70.3%)	0.4879
RTK/RAS Alterations Absent	21 (23.1%)	280 (30.5%)		12 (24.0%)	194 (29.7%)	

## Data Availability

The data presented in this study are openly available in cBioPortal at https://www.cbioportal.org (accessed on 10 September 2025) and https://genie.cbioportal.org/login (accessed on 10 September 2025). Analytical resources are available through the GitHub repository https://github.com/Velazquez-Villarreal-Lab/AI-TP53 (accessed on 10 September 2025) to promote transparency and reproducibility. Additional data can be provided by the authors upon reasonable request.

## Supplementary Materials

The following supporting information can be downloaded at: https://www.mdpi.com/article/10.3390/cancers18020239/s1, Figure S1: Schematic Overview of the AI-HOPE–Enabled Analytical Workflow for RTK–RAS Biomarker Discovery; Figure S2: AI-enabled cohort definition and survival stratification of early-onset colorectal cancer (EOCRC) non-Hispanic White (NHW) patients not treated with FOLFOX based on RTK-RAS pathway status; Figure S3: AI-driven case-control composition and comparative analysis of pathway-defined cohorts in early-onset colorectal cancer (EOCRC); Figure S4: Systematic AI-assisted comparison of clinical and genomic features between early-onset colorectal cancer (EOCRC) Hispanic/Latino (H/L) and Non-Hispanic White (NHW) cohorts; Figure S5: AI-assisted case-control evaluation of BRAF mutation prevalence in FOLFOX-treated EOCRC by ancestry; Table S1: Comparison of RTK-RAS pathway alteration frequencies in early-onset Hispanic/Latino (H/L) colorectal cancer patients treated with FOLFOX versus not treated with FOLFOX; Table S2: Comparison of RTK-RAS pathway alteration frequencies in late-onset Hispanic/Latino (H/L) colorectal cancer patients treated with FOLFOX versus not treated with FOLFOX; Table S3: Comparison of RTK-RAS pathway alteration frequencies between early-onset and late-onset Hispanic/Latino (H/L) colorectal cancer patients treated with FOLFOX; Table S4: Comparison of RTK-RAS pathway alteration frequencies between early-onset and late-onset Hispanic/Latino (H/L) colorectal cancer patients not treated with FOLFOX; Table S5: Comparison of RTK-RAS pathway alteration frequencies in early-onset Non-Hispanic White (NHW) colorectal cancer patients treated with FOLFOX versus not treated with FOLFOX; Table S6: Comparison of RTK-RAS pathway alteration frequencies in late-onset Non-Hispanic White (NHW) colorectal cancer patients treated with FOLFOX versus not treated with FOLFOX; Table S7: Comparison of RTK-RAS pathway alteration frequencies between early-onset and late-onset Non-Hispanic White (NHW) colorectal cancer patients treated with FOLFOX; Table S8: Comparison of RTK-RAS pathway alteration frequencies between early-onset and late-onset Non-Hispanic White (NHW) colorectal cancer patients not treated with FOLFOX; Table S9: Comparison of RTK-RAS pathway alteration frequencies between early-onset Hispanic/Latino (H/L) and early-onset Non-Hispanic White (NHW) colorectal cancer patients treated with FOLFOX; Table S10: Comparison of RTK-RAS pathway alteration frequencies between early-onset Hispanic/Latino (H/L) and early-onset Non-Hispanic White (NHW) colorectal cancer patients not treated with FOLFOX; Table S11: Comparison of RTK-RAS pathway alteration frequencies between late-onset Hispanic/Latino (H/L) and late-onset Non-Hispanic White (NHW) colorectal cancer patients treated with FOLFOX; Table S12: Comparison of RTK-RAS pathway alteration frequencies between late-onset Hispanic/Latino (H/L) and late-onset Non-Hispanic White (NHW) colorectal cancer patients not treated with FOLFOX; Table S13: Spectrum of RTK-RAS pathway variant classes across colorectal cancer subgroups defined by ancestry, age at diagnosis, and FOLFOX exposure; Table S14: Distribution of Colorectal Cancer Cases by Data Source, Ancestry, Age at Onset, and FOLFOX Treatment Status.

## References

[1] Ciardiello F., Ciardiello D., Martini G., Napolitano S., Tabernero J., Cervantes A. (2022). Clinical management of metastatic colorectal cancer in the era of precision medicine. CA Cancer J. Clin..

[2] Ciombor K.K., Wu C., Goldberg R.M. (2015). Recent therapeutic advances in the treatment of colorectal cancer. Annu. Rev. Med..

[3] Gmeiner W.H. (2024). Recent advances in therapeutic strategies to improve colorectal cancer treatment. Cancers.

[4] Tran N.H., Cavalcante L.L., Lubner S.J., Mulkerin D.L., LoConte N.K., Clipson L., Matkowskyj K.A., Deming D.A. (2015). Precision medicine in colorectal cancer: The molecular profile alters treatment strategies. Ther. Adv. Med. Oncol..

[5] Abudalo R., Alqudah A., Alnajjar R., Abudalo R., Abuqamar A., Oqal M., Qnais E. (2025). KRAS/NRAS/BRAF mutational profile and association with clinicopathological characteristics in patients with metastatic colorectal cancer. Oncol. Lett..

[6] Douillard J.Y., Oliner K.S., Siena S., Tabernero J., Burkes R., Barugel M., Humblet Y., Bodoky G., Cunningham D., Jassem J. (2013). Panitumumab–FOLFOX4 treatment and RAS mutations in colorectal cancer. N. Engl. J. Med..

[7] Douillard J.Y., Siena S., Cassidy J., Tabernero J., Burkes R., Barugel M., Humblet Y., Bodoky G., Cunningham D., Jassem J. (2010). Randomized phase III trial of panitumumab with FOLFOX4 versus FOLFOX4 alone as first-line treatment of metastatic colorectal cancer (PRIME study). J. Clin. Oncol..

[8] Giordano G., Parcesepe P., Bruno G., Piscazzi A., Lizzi V., Remo A., Pancione M., D’Andrea M.R., De Santis E., Coppola L. (2021). Evidence-based second-line treatment in RAS wild-type/mutated metastatic colorectal cancer in the precision medicine era. Int. J. Mol. Sci..

[9] Lai E., Liscia N., Donisi C., Mariani S., Tolu S., Pretta A., Persano M., Pinna G., Balconi F., Pireddu A. (2020). Molecular biology–driven treatment for metastatic colorectal cancer. Cancers.

[10] Peeters M., Price T. (2012). Biologic therapies in the metastatic colorectal cancer treatment continuum. Cancer Treat. Rev..

[11] Sakata S., Larson D.W. (2022). Targeted therapy for colorectal cancer. Surg. Oncol. Clin. N. Am..

[12] Bokemeyer C., Bondarenko I., Hartmann J.T., de Braud F., Schuch G., Zubel A., Celik I., Schlichting M., Koralewski P. (2011). Efficacy according to biomarker status of cetuximab plus FOLFOX4 as first-line treatment of metastatic colorectal cancer (OPUS study). Ann. Oncol..

[13] Bokemeyer C., Bondarenko I., Makhson A., Hartmann J.T., Aparicio J., de Braud F., Donea S., Ludwig H., Schuch G., Stroh C. (2009). Fluorouracil, leucovorin, and oxaliplatin with or without cetuximab in first-line metastatic colorectal cancer. J. Clin. Oncol..

[14] Bokemeyer C., Köhne C.H., Ciardiello F., Lenz H.J., Heinemann V., Klinkhardt U., Beier F., Duecker K., van Krieken J.H., Tejpar S. (2015). FOLFOX4 plus cetuximab treatment and RAS mutations in colorectal cancer. Eur. J. Cancer.

[15] Fu S., Ma J., Cai C., Tan J., Deng X., Shen H., Zeng S., Chen Y., Han Y. (2025). Precision immune regulation in KRAS-mutated cancers. J. Exp. Clin. Cancer Res..

[16] Janssens K., Lambrechts C., Geerinckx B., Op de Beeck K., Van Camp G., Oliveres H., Prenen H., Vandamme T., Peeters M. (2023). New developments in treating RAS-mutated metastatic colorectal cancer. Curr. Treat. Options Oncol..

[17] Sütcüoğlu O., Yıldırım H.Ç., Almuradova E., Günenç D., Yalçın Ş. (2025). RAS mutations in advanced colorectal cancer. Medicina.

[18] Takeda M., Yoshida S., Inoue T., Sekido Y., Hata T., Hamabe A., Ogino T., Miyoshi N., Uemura M., Yamamoto H. (2025). Role of KRAS mutations in colorectal cancer. Cancers.

[19] Zhu G., Pei L., Xia H., Tang Q., Bi F. (2021). Role of oncogenic KRAS in colorectal cancer. Mol. Cancer.

[20] Chang M.H., Lee I.K., Si Y., Lee K.S., Woo I.S., Byun J.H. (2011). Clinical impact of KRAS mutation in colorectal cancer treated with adjuvant FOLFOX. Cancer Chemother. Pharmacol..

[21] Chua W., Goldstein D., Lee C.K., Dhillon H., Michael M., Mitchell P., Clarke S.J., Iacopetta B. (2009). Molecular markers of response and toxicity to FOLFOX chemotherapy in metastatic colorectal cancer. Br. J. Cancer.

[22] Deng Y., Wang L., Tan S., Kim G.P., Dou R., Chen D., Cai Y., Fu X., Wang L., Zhu J. (2015). KRAS as a predictor of poor prognosis and benefit from postoperative FOLFOX chemotherapy in stage II and III colorectal cancer. Mol. Oncol..

[23] Lee D.W., Han S.W., Cha Y., Bae J.M., Kim H.P., Lyu J., Han H., Kim H., Jang H., Bang D. (2019). Association of pathway mutation with survival after recurrence in colorectal cancer patients treated with adjuvant fluoropyrimidine and oxaliplatin chemotherapy. BMC Cancer.

[24] Lee D.W., Kim K.J., Han S.W., Lee H.J., Rhee Y.Y., Bae J.M., Cho N.Y., Lee K.H., Kim T.Y., Oh D.Y. (2015). KRAS mutation is associated with worse prognosis in stage III or high-risk stage II colon cancer treated with adjuvant FOLFOX. Ann. Surg. Oncol..

[25] Lin Y.L., Liau J.Y., Yu S.C., Ou D.L., Lin L.I., Tseng L.H., Chang Y.L., Yeh K.H., Cheng A.L. (2012). KRAS mutation predicts oxaliplatin sensitivity in colon cancer cells. PLoS ONE.

[26] Park H.E., Yoo S.Y., Cho N.Y., Bae J.M., Han S.W., Lee H.S., Park K.J., Kim T.Y., Kang G.H. (2021). Tumor microenvironment–adjusted prognostic implications of KRAS mutation subtype in stage III colorectal cancer treated with adjuvant FOLFOX. Sci. Rep..

[27] Kim S.H., Shin S.J., Lee K.Y., Kim H., Kim T.I., Kang D.R., Hur H., Min B.S., Kim N.K., Chung H.C. (2013). Prognostic value of mucinous histology depends on microsatellite instability status in stage III colon cancer treated with adjuvant FOLFOX chemotherapy. Ann. Surg. Oncol..

[28] Park S.M., Choi S.B., Lee Y.S., Lee I.K. (2021). Predictive value of KRAS mutation and ERCC1 protein overexpression in colorectal cancer patients receiving FOLFOX. Asian J. Surg..

[29] Sharma N., Saifo M., Tamaskar I.R., Bhuvaneswari R., Mashtare T., Fakih M. (2010). KRAS status and clinical outcome in metastatic colorectal cancer treated with first-line FOLFOX chemotherapy. J. Gastrointest. Oncol..

[30] Formica V., Sera F., Cremolini C., Riondino S., Morelli C., Arkenau H.T., Roselli M. (2022). KRAS and BRAF mutations in stage II and III colon cancer: A systematic review and meta-analysis. J. Natl. Cancer Inst..

[31] Zocche D.M., Ramirez C., Fontao F.M., Costa L.D., Redal M.A. (2015). Global impact of KRAS mutation patterns in FOLFOX-treated metastatic colorectal cancer. Front. Genet..

[32] Hoshida T., Tsubaki M., Takeda T., Asano R., Choi I.H., Takimoto K., Inukai A., Imano M., Tanabe K., Nagai N. (2025). Oxaliplatin and 5-fluorouracil promote epithelial–mesenchymal transition via KRAS/ERK/NF-κB activation in KRAS-mutated colon cancer cells. Mol. Cell. Biochem..

[33] Diaz F.C., Waldrup B., Carranza F.G., Manjarrez S., Velazquez-Villarreal E. (2025). Artificial intelligence–guided molecular determinants of PI3K pathway alterations in early-onset colorectal cancer receiving FOLFOX. Biomedicines.

[34] Diaz F.C., Waldrup B., Carranza F.G., Manjarrez S., Velazquez-Villarreal E. (2025). AI-enhanced precision medicine reveals prognostic impact of TGF-β pathway alterations in FOLFOX-treated early-onset colorectal cancer. Int. J. Mol. Sci..

[35] Diaz F.C., Waldrup B., Carranza F.G., Manjarrez S., Velazquez-Villarreal E. (2025). Precision oncology insights into WNT pathway alterations in FOLFOX-treated early-onset colorectal cancer. Cancers.

[36] Monge C., Waldrup B., Carranza F.G., Velazquez-Villarreal E. (2025). Molecular heterogeneity in early-onset colorectal cancer: Pathway-specific insights in high-risk populations. Cancers.

[37] Monge C., Waldrup B., Carranza F.G., Velazquez-Villarreal E. (2025). Molecular alterations in TP53, WNT, PI3K, and RTK–RAS pathways in gastric cancer across ethnically heterogeneous cohorts. Cancers.

[38] Yang E.W., Velazquez-Villarreal E. (2025). AI-HOPE: A conversational AI for enhanced clinical and genomic data integration in precision medicine. Bioinformatics.

[39] Yang E.W., Waldrup B., Velazquez-Villarreal E. (2025). Precision oncology through dialogue: AI-HOPE-RTK–RAS integrates clinical and genomic insights in colorectal cancer. Biomedicines.

[40] Wei L., Chen J., Wen J., Wu D., Ma X., Chen Z., Huang J. (2020). Efficacy of oxaliplatin/5-fluorouracil/capecitabine–cetuximab combination therapy and effects on KRAS mutations in advanced colorectal cancer. Med. Sci. Monit..

[41] Smith J.C., Brooks L., Hoff P.M., McWalter G., Dearden S., Morgan S.R., Wilson D., Robertson J.D., Jürgensmeier J.M. (2013). KRAS mutations and clinical outcome in metastatic colorectal cancer. Eur. J. Cancer.

[42] Ryu W.J., Lee J.E., Cho Y.H., Lee G., Seo M.K., Lee S.K., Hwang J.H., Min D.S., Noh S.H., Paik S. (2019). Therapeutic strategy for chemotherapy-resistant gastric cancer via destabilization of β-catenin and RAS. Cancers.

[43] Rasmy A., Fayed A., Omar A., Fahmy N. (2019). Effect of KRAS mutational status on disease behavior and treatment outcome in metastatic colorectal cancer. J. Gastrointest. Oncol..

[44] Pirvu E.E., Severin E., Niţă I., Toma Ş.A. (2023). Impact of RAS mutation on colorectal cancer treatment strategies. Med. Pharm. Rep..

[45] Pathak S., Banerjee A., Marotta F., Gopinath M., Murugesan R., Zhang H., Girigoswami A., Sollano J., Sun X.F. (2017). Comparative efficacy of bevacizumab, panitumumab, and cetuximab with FOLFOX4 in KRAS-mutated colorectal cancer. Oncotarget.

[46] Ottaiano A., Normanno N., Facchini S., Cassata A., Nappi A., Romano C., Silvestro L., De Stefano A., Rachiglio A.M., Roma C. (2020). Prognostic value of RAS mutations in metastatic colorectal cancer (STORIA analysis). Cancers.

[47] Kim M.J., Lee H.S., Kim J.H., Kim Y.J., Kwon J.H., Lee J.O., Bang S.M., Park K.U., Kim D.W., Kang S.B. (2012). Metastatic patterns according to KRAS status in colorectal cancer. BMC Cancer.

[48] Kawamoto Y., Tsuchihara K., Yoshino T., Ogasawara N., Kojima M., Takahashi M., Ochiai A., Bando H., Fuse N., Tahara M. (2012). KRAS mutations in primary tumors and post-FOLFOX metastases in colorectal cancer. Br. J. Cancer.

[49] Kang S., Lee M.W., Song I.C., Lee H.J., Yun H.J., Jo D.Y., Kim J.S., Kwon J.H., Kim J.Y., Lee K.H. (2023). Maintenance therapy after first-line FOLFOX according to RAS or BRAFV600E status in metastatic colorectal cancer. J. Cancer Res. Clin. Oncol..

[50] Del Rio M., Mollevi C., Bibeau F., Vie N., Selves J., Emile J.F., Roger P., Gongora C., Robert J., Tubiana-Mathieu N. (2017). Molecular subtypes of metastatic colorectal cancer and response to irinotecan-based therapies. Eur. J. Cancer.

[51] Cerami E., Gao J., Dogrusoz U., Gross B.E., Sumer S.O., Aksoy B.A., Jacobsen A., Byrne C.J., Heuer M.L., Larsson E. (2012). The cBio cancer genomics portal. Cancer Discov..

[52] Cancer Genome Atlas Research Network (2013). The Cancer Genome Atlas Pan-Cancer analysis project. Nat. Genet..

[53] Jee J., Fong C., Pichotta K., Tran T.N., Luthra A., Waters M., Fu C., Altoe M., Liu S.Y., Maron S.B. (2024). Automated real-world data integration improves cancer outcome prediction. Nature.

[54] American Association for Cancer Research AACR Project GENIE Biopharma Collaborative (BPC). https://www.aacr.org/professionals/research/aacr-project-genie/bpc/.

