# Supplementary material for: Prognostic Impact of RTK–RAS Alterations in FOLFOX-Treated Early-Onset Colorectal Cancer Revealed by Artificial Intelligence-Driven Precision Oncology
Source: Cancers (Basel). 2026 Jan 13;18(2):239. doi: 10.3390/cancers18020239 (PMC12838724; doi:10.3390/cancers18020239)
Supplement: Supplementary file 1 [file cancers-18-00239-s001.zip › cancers-4071495-supplementary.pdf]

Supplementary Materials:

Table S1. Comparison of RTK-RAS pathway alteration frequencies in early-onset Hispanic/Latino (H/L) colorectal cancer patients treated with FOLFOX versus not treated with FOLFOX.

RTK/RAS Pathway			
**Gene**	**Early-Onset Hispanic/Latino Treated with FOLFOX n (%)**	**Early-Onset Hispanic/Latino Not Treated with FOLFOX n (%)**	**p-value**
**EGFR Mutation**			
Present	1 (1.4%)	3 (5.8%)	0.3067
Absent	72 (98.6%)	49 (94.2%)	
**ERBB2 Mutation**			
Present	2 (2.7%)	8 (15.4%)	**0.01626**
Absent	71 (97.3%)	44 (84.6%)	
**ERBB3 Mutation**			
Present	4 (5.5%)	3 (5.8%)	1
Absent	69 (94.5%)	49 (94.2%)	
**ERBB4 Mutation**			
Present	3 (4.1%)	7 (13.5%)	0.09163
Absent	70 (95.9%)	45 (86.5%)	
**FGFR1 Mutation**			
Present	0 (0.0%)	2 (3.8%)	0.1711
Absent	73 (100.0%)	50 (96.2%)	
**FGFR2 Mutation**			
Present	0 (0.0%)	4 (7.7%)	0.02793
Absent	73 (100.0%)	48 (92.3%)	
**FGFR3 Mutation**			
Present	1 (1.4%)	2 (3.8%)	0.5699
Absent	72 (98.6%)	50 (96.2%)	
**FGFR4 Mutation**			
Present	1 (1.4%)	1 (1.9%)	1
Absent	72 (98.6%)	51 (98.1%)	
**KRAS Mutation**			
Present	30 (41.1%)	18 (34.6%)	0.5839
Absent	43 (58.9%)	34 (65.4%)	
**NRAS Mutation**			
Present	2 (2.7%)	3 (5.8%)	0.6484
Absent	71 (97.3%)	49 (94.2%)	
**HRAS Mutation**			
Present	1 (1.4%)	1 (1.9%)	1
Absent	72 (98.6%)	51 (98.1%)	
**BRAF Mutation**			
Present	3 (4.1%)	4 (7.7%)	0.4486
Absent	70 (95.9%)	48 (92.3%)	
**MAP2K1 Mutation**			
Present	2 (2.7%)	3 (5.8%)	0.6484
Absent	71 (97.3%)	49 (94.2%)	
**MAP2K2 Mutation**			
Present	1 (1.4%)	1 (1.9%)	1
Absent	72 (98.6%)	51 (98.1%)	
**MAPK1 Mutation**			
Present	0 (0.0%)	2 (3.8%)	0.1711
Absent	73 (100.0%)	50 (96.2%)	
**MAPK3 Mutation**			
Present	1 (1.4%)	3 (5.8%)	0.3067
Absent	72 (98.6%)	49 (94.2%)	
**SOS1 Mutation**			
Present	0 (0.0%)	1 (1.9%)	0.416
Absent	73 (100.0%)	51 (98.1%)	
**MET Mutation**			
Present	1 (1.4%)	0 (0.0%)	1
Absent	72 (98.6%)	52 (100.0%)	
**PDGFRA Mutation**			
Present	2 (2.7%)	5 (9.6%)	0.1264
Absent	71 (97.3%)	47 (90.4%)	
**KIT Mutation**			
Present	1 (1.4%)	3 (5.8%)	0.3067
Absent	72 (98.6%)	49 (94.2%)	
**IGF1R Mutation**			
Present	1 (1.4%)	4 (7.7%)	0.1608
Absent	72 (98.6%)	48 (92.3%)	
**RET Mutation**			
Present	1 (1.4%)	1 (1.9%)	1
Absent	72 (98.6%)	51 (98.1%)	
**ROS1 Mutation**			
Present	2 (2.7%)	4 (7.7%)	0.2328
Absent	71 (97.3%)	48 (92.3%)	
**ALK Mutation**			
Present	2 (2.7%)	2 (3.8%)	1
Absent	71 (97.3%)	50 (96.2%)	
**FLT3 Mutation**			
Present	1 (1.4%)	4 (7.7%)	0.1608
Absent	72 (98.6%)	48 (92.3%)	
**NTRK1 Mutation**			
Present	1 (1.4%)	2 (3.8%)	0.5699
Absent	72 (98.6%)	50 (96.2%)	
**NTRK2 Mutation**			
Present	1 (1.4%)	2 (3.8%)	0.5699
Absent	72 (98.6%)	50 (96.2%)	
**CBL Mutation**			
Present	2 (2.7%)	5 (9.6%)	0.1264
Absent	71 (97.3%)	47 (90.4%)	
**ERRFI1 Mutation**			
Present	0 (0.0%)	0 (0.0%)	1
Absent	73 (100.0%)	52 (100.0%)	
**NF1 Mutation**			
Present	3 (4.1%)	10 (19.2%)	**0.01437**
Absent	70 (95.9%)	42 (80.8%)	
**RASA1 Mutation**			
Present	1 (1.4%)	3 (5.8%)	0.3067
Absent	72 (98.6%)	49 (94.2%)	
**PTPN11 Mutation**			
Present	0 (0.0%)	0 (0.0%)	1
Absent	73 (100.0%)	52 (100.0%)	
**RIT1 Mutation**			
Present	1 (1.4%)	1 (1.9%)	1
Absent	72 (98.6%)	51 (98.1%)	
**ARAF Mutation**			
Present	0 (0.0%)	1 (1.9%)	0.416
Absent	73 (100.0%)	51 (98.1%)	
**RAF1 Mutation**			
Present	1 (1.4%)	2 (3.8%)	0.5699
Absent	72 (98.6%)	50 (96.2%)	
**RAC1 Mutation**			
Present	0 (0.0%)	0 (0.0%)	1
Absent	73 (100.0%)	52 (100.0%)	

Table S2. Comparison of RTK-RAS pathway alteration frequencies in late-onset Hispanic/Latino (H/L) colorectal cancer patients treated with FOLFOX versus not treated with FOLFOX.

RTK/RAS Pathway			
**Gene**	**Late-Onset Hispanic/Latino Treated with FOLFOX n (%)**	**Late-Onset Hispanic/Latino Not Treated with FOLFOX n (%)**	**p-value**
**EGFR Mutation**			
Present	1 (1.1%)	3 (6.0%)	0.1276
Absent	90 (98.9%)	47 (94.0%)	
**ERBB2 Mutation**			
Present	2 (2.2%)	5 (10.0%)	0.09692
Absent	89 (97.8%)	45 (90.0%)	
**ERBB3 Mutation**			
Present	1 (1.1%)	3 (6.0%)	0.1276
Absent	90 (98.9%)	47 (94.0%)	
**ERBB4 Mutation**			
Present	3 (3.3%)	3 (6.0%)	0.6659
Absent	88 (96.7%)	47 (94.0%)	
**FGFR1 Mutation**			
Present	0 (0.0%)	0 (0.0%)	1
Absent	91 (100.0%)	50 (100.0%)	
**FGFR2 Mutation**			
Present	0 (0.0%)	1 (2.0%)	0.3546
Absent	91 (100.0%)	49 (98.0%)	
**FGFR3 Mutation**			
Present	3 (3.3%)	2 (4.0%)	1
Absent	88 (96.7%)	48 (96.0%)	
**FGFR4 Mutation**			
Present	1 (1.1%)	1 (2.0%)	1
Absent	90 (98.9%)	49 (98.0%)	
**KRAS Mutation**			
Present	39 (42.9%)	20 (40.0%)	0.8803
Absent	52 (57.1%)	30 (60.0%)	
**NRAS Mutation**			
Present	6 (6.6%)	3 (6.0%)	1
Absent	85 (93.4%)	47 (94.0%)	
**HRAS Mutation**			
Present	0 (0.0%)	1 (2.0%)	0.3546
Absent	91 (100.0%)	49 (98.0%)	
**BRAF Mutation**			
Present	15 (16.5%)	8 (16.0%)	1
Absent	76 (83.5%)	42 (84.0%)	
**MAP2K1 Mutation**			
Present	0 (0.0%)	0 (0.0%)	1
Absent	91 (100.0%)	50 (100.0%)	
**MAP2K2 Mutation**			
Present	2 (2.2%)	1 (2.0%)	1
Absent	89 (97.8%)	49 (98.0%)	
**MAPK1 Mutation**			
Present	0 (0.0%)	0 (0.0%)	1
Absent	91 (100.0%)	50 (100.0%)	
**MAPK3 Mutation**			
Present	1 (1.1%)	0 (0.0%)	1
Absent	90 (98.9%)	50 (100.0%)	
**SOS1 Mutation**			
Present	3 (3.3%)	0 (0.0%)	0.5523
Absent	88 (96.7%)	50 (100.0%)	
**MET Mutation**			
Present	4 (4.4%)	2 (4.0%)	1
Absent	87 (95.6%)	48 (96.0%)	
**PDGFRA Mutation**			
Present	0 (0.0%)	0 (0.0%)	1
Absent	91 (100.0%)	50 (100.0%)	
**KIT Mutation**			
Present	1 (1.1%)	0 (0.0%)	1
Absent	90 (98.9%)	50 (100.0%)	
**IGF1R Mutation**			
Present	1 (1.1%)	3 (6.0%)	0.1276
Absent	90 (98.9%)	47 (94.0%)	
**RET Mutation**			
Present	3 (3.3%)	2 (4.0%)	1
Absent	88 (96.7%)	48 (96.0%)	
**ROS1 Mutation**			
Present	6 (6.6%)	2 (4.0%)	0.712
Absent	85 (93.4%)	48 (96.0%)	
**ALK Mutation**			
Present	4 (4.4%)	2 (4.0%)	1
Absent	87 (95.6%)	48 (96.0%)	
**FLT3 Mutation**			
Present	2 (2.2%)	3 (6.0%)	0.3466
Absent	89 (97.8%)	47 (94.0%)	
**NTRK1 Mutation**			
Present	2 (2.2%)	2 (4.0%)	0.615
Absent	89 (97.8%)	48 (96.0%)	
**NTRK2 Mutation**			
Present	0 (0.0%)	3 (6.0%)	**0.04286**
Absent	91 (100.0%)	47 (94.0%)	
**CBL Mutation**			
Present	0 (0.0%)	1 (2.0%)	1
Absent	91 (100.0%)	49 (98.0%)	
**ERRFI1 Mutation**			
Present	0 (0.0%)	0 (0.0%)	1
Absent	91 (100.0%)	50 (100.0%)	
**NF1 Mutation**			
Present	2 (2.2%)	2 (4.0%)	0.615
Absent	89 (97.8%)	48 (96.0%)	
**RASA1 Mutation**			
Present	4 (4.4%)	1 (2.0%)	0.6557
Absent	87 (95.6%)	49 (98.0%)	
**PTPN11 Mutation**			
Present	2 (2.2%)	1 (2.0%)	1
Absent	89 (97.8%)	49 (98.0%)	
**RIT1 Mutation**			
Present	1 (1.1%)	0 (0.0%)	1
Absent	90 (98.9%)	50 (100.0%)	
**ARAF Mutation**			
Present	3 (3.3%)	2 (4.0%)	1
Absent	88 (96.7%)	48 (96.0%)	
**RAF1 Mutation**			
Present	2 (2.2%)	1 (2.0%)	1
Absent	89 (97.8%)	49 (98.0%)	
**RAC1 Mutation**			
Present	0 (0.0%)	0 (0.0%)	1
Absent	91 (100.0%)	50 (100.0%)	

Table S3. Comparison of RTK-RAS pathway alteration frequencies between early-onset and late-onset Hispanic/Latino (H/L) colorectal cancer patients treated with FOLFOX.

RTK/RAS Pathway			
**Gene**	**Early-Onset Hispanic/Latino Treated with FOLFOX n (%)**	**Late-Onset Hispanic/Latino Treated with FOLFOX n (%)**	**p-value**
**EGFR Mutation**			
Present	1 (1.4%)	1 (1.1%)	1
Absent	72 (98.6%)	90 (98.9%)	
**ERBB2 Mutation**			
Present	2 (2.7%)	2 (2.2%)	1
Absent	71 (97.3%)	89 (97.8%)	
**ERBB3 Mutation**			
Present	4 (5.5%)	1 (1.1%)	0.1727
Absent	69 (94.5%)	90 (98.9%)	
**ERBB4 Mutation**			
Present	3 (4.1%)	3 (3.3%)	1
Absent	70 (95.9%)	88 (96.7%)	
**FGFR1 Mutation**			
Present	0 (0.0%)	0 (0.0%)	1
Absent	73 (100.0%)	91 (100.0%)	
**FGFR2 Mutation**			
Present	0 (0.0%)	0 (0.0%)	1
Absent	73 (100.0%)	91 (100.0%)	
**FGFR3 Mutation**			
Present	1 (1.4%)	3 (3.3%)	0.6296
Absent	72 (98.6%)	88 (96.7%)	
**FGFR4 Mutation**			
Present	1 (1.4%)	1 (1.1%)	1
Absent	72 (98.6%)	90 (98.9%)	
**KRAS Mutation**			
Present	30 (41.1%)	39 (42.9%)	0.9458
Absent	43 (58.9%)	52 (57.1%)	
**NRAS Mutation**			
Present	2 (2.7%)	6 (6.6%)	0.3012
Absent	71 (97.3%)	85 (93.4%)	
**HRAS Mutation**			
Present	1 (1.4%)	0 (0.0%)	0.4451
Absent	72 (98.6%)	91 (100.0%)	
**BRAF Mutation**			
Present	3 (4.1%)	15 (16.5%)	**0.01229**
Absent	70 (95.9%)	76 (83.5%)	
**MAP2K1 Mutation**			
Present	2 (2.7%)	0 (0.0%)	0.1966
Absent	71 (97.3%)	91 (100.0%)	
**MAP2K2 Mutation**			
Present	1 (1.4%)	2 (2.2%)	1
Absent	72 (98.6%)	89 (97.8%)	
**MAPK1 Mutation**			
Present	0 (0.0%)	0 (0.0%)	1
Absent	73 (100.0%)	91 (100.0%)	
**MAPK3 Mutation**			
Present	1 (1.4%)	1 (1.1%)	1
Absent	72 (98.6%)	90 (98.9%)	
**SOS1 Mutation**			
Present	0 (0.0%)	3 (3.3%)	0.2545
Absent	73 (100.0%)	88 (96.7%)	
**MET Mutation**			
Present	1 (1.4%)	4 (4.4%)	0.3826
Absent	72 (98.6%)	87 (95.6%)	
**PDGFRA Mutation**			
Present	2 (2.7%)	0 (0.0%)	0.1966
Absent	71 (97.3%)	91 (100.0%)	
**KIT Mutation**			
Present	1 (1.4%)	1 (1.1%)	1
Absent	72 (98.6%)	90 (98.9%)	
**IGF1R Mutation**			
Present	1 (1.4%)	1 (1.1%)	1
Absent	72 (98.6%)	90 (98.9%)	
**RET Mutation**			
Present	1 (1.4%)	3 (3.3%)	0.6296
Absent	72 (98.6%)	88 (96.7%)	
**ROS1 Mutation**			
Present	2 (2.7%)	6 (6.6%)	0.3012
Absent	71 (97.3%)	85 (93.4%)	
**ALK Mutation**			
Present	2 (2.7%)	4 (4.4%)	0.6933
Absent	71 (97.3%)	87 (95.6%)	
**FLT3 Mutation**			
Present	1 (1.4%)	2 (2.2%)	1
Absent	72 (98.6%)	89 (97.8%)	
**NTRK1 Mutation**			
Present	1 (1.4%)	2 (2.2%)	1
Absent	72 (98.6%)	89 (97.8%)	
**NTRK2 Mutation**			
Present	1 (1.4%)	0 (0.0%)	0.4451
Absent	72 (98.6%)	91 (100.0%)	
**CBL Mutation**			
Present	2 (2.7%)	0 (0.0%)	0.1966
Absent	71 (97.3%)	91 (100.0%)	
**ERRFI1 Mutation**			
Present	0 (0.0%)	0 (0.0%)	1
Absent	73 (100.0%)	91 (100.0%)	
**NF1 Mutation**			
Present	3 (4.1%)	2 (2.2%)	0.6566
Absent	70 (95.9%)	89 (97.8%)	
**RASA1 Mutation**			
Present	1 (1.4%)	4 (4.4%)	0.3826
Absent	72 (98.6%)	87 (95.6%)	
**PTPN11 Mutation**			
Present	0 (0.0%)	2 (2.2%)	0.503
Absent	73 (100.0%)	89 (97.8%)	
**RIT1 Mutation**			
Present	1 (1.4%)	1 (1.1%)	1
Absent	72 (98.6%)	90 (98.9%)	
**ARAF Mutation**			
Present	0 (0.0%)	3 (3.3%)	0.2545
Absent	73 (100.0%)	88 (96.7%)	
**RAF1 Mutation**			
Present	1 (1.4%)	2 (2.2%)	1
Absent	72 (98.6%)	89 (97.8%)	
**RAC1 Mutation**			
Present	0 (0.0%)	0 (0.0%)	1
Absent	73 (100.0%)	91 (100.0%)	

Table S4. Comparison of RTK-RAS pathway alteration frequencies between early-onset and late-onset Hispanic/Latino (H/L) colorectal cancer patients not treated with FOLFOX.

RTK/RAS Pathway			
Gene	Early-Onset Hispanic/Latino Not Treated with FOLFOX n (%)	Late-Onset Hispanic/Latino Not Treated with FOLFOX n (%)	p-value
**EGFR Mutation**			
Present	3 (5.8%)	3 (6.0%)	1
Absent	49 (94.2%)	47 (94.0%)	
**ERBB2 Mutation**			
Present	8 (15.4%)	5 (10.0%)	0.6043
Absent	44 (84.6%)	45 (90.0%)	
**ERBB3 Mutation**			
Present	3 (5.8%)	3 (6.0%)	1
Absent	49 (94.2%)	47 (94.0%)	
**ERBB4 Mutation**			
Present	7 (13.5%)	3 (6.0%)	0.3194
Absent	45 (86.5%)	47 (94.0%)	
**FGFR1 Mutation**			
Present	2 (3.8%)	0 (0.0%)	0.4952
Absent	50 (96.2%)	50 (100.0%)	
**FGFR2 Mutation**			
Present	4 (7.7%)	1 (2.0%)	0.3629
Absent	48 (92.3%)	49 (98.0%)	
**FGFR3 Mutation**			
Present	2 (3.8%)	2 (4.0%)	1
Absent	50 (96.2%)	48 (96.0%)	
**FGFR4 Mutation**			
Present	1 (1.9%)	1 (2.0%)	1
Absent	51 (98.1%)	49 (98.0%)	
**KRAS Mutation**			
Present	18 (34.6%)	20 (40.0%)	0.7208
Absent	34 (65.4%)	30 (60.0%)	
**NRAS Mutation**			
Present	3 (5.8%)	3 (6.0%)	1
Absent	49 (94.2%)	47 (94.0%)	
**HRAS Mutation**			
Present	1 (1.9%)	1 (2.0%)	1
Absent	51 (98.1%)	49 (98.0%)	
**BRAF Mutation**			
Present	4 (7.7%)	8 (16.0%)	0.2303
Absent	48 (92.3%)	42 (84.0%)	
**MAP2K1 Mutation**			
Present	3 (5.8%)	0 (0.0%)	0.2429
Absent	49 (94.2%)	50 (100.0%)	
**MAP2K2 Mutation**			
Present	1 (1.9%)	1 (2.0%)	1
Absent	51 (98.1%)	49 (98.0%)	
**MAPK1 Mutation**			
Present	2 (3.8%)	0 (0.0%)	0.4952
Absent	50 (96.2%)	50 (100.0%)	
**MAPK3 Mutation**			
Present	3 (5.8%)	0 (0.0%)	0.2429
Absent	49 (94.2%)	50 (100.0%)	
**SOS1 Mutation**			
Present	1 (1.9%)	0 (0.0%)	1
Absent	51 (98.1%)	50 (100.0%)	
**MET Mutation**			
Present	0 (0.0%)	2 (4.0%)	0.2378
Absent	52 (100.0%)	48 (96.0%)	
**PDGFRA Mutation**			
Present	5 (9.6%)	0 (0.0%)	0.05664
Absent	47 (90.4%)	50 (100.0%)	
**KIT Mutation**			
Present	3 (5.8%)	0 (0.0%)	0.2429
Absent	49 (94.2%)	50 (100.0%)	
**IGF1R Mutation**			
Present	4 (7.7%)	3 (6.0%)	1
Absent	48 (92.3%)	47 (94.0%)	
**RET Mutation**			
Present	1 (1.9%)	2 (4.0%)	0.6139
Absent	51 (98.1%)	48 (96.0%)	
**ROS1 Mutation**			
Present	4 (7.7%)	2 (4.0%)	0.6783
Absent	48 (92.3%)	48 (96.0%)	
**ALK Mutation**			
Present	2 (3.8%)	2 (4.0%)	1
Absent	50 (96.2%)	48 (96.0%)	
**FLT3 Mutation**			
Present	4 (7.7%)	3 (6.0%)	1
Absent	48 (92.3%)	47 (94.0%)	
**NTRK1 Mutation**			
Present	2 (3.8%)	2 (4.0%)	1
Absent	50 (96.2%)	48 (96.0%)	
**NTRK2 Mutation**			
Present	2 (3.8%)	3 (6.0%)	0.645
Absent	50 (96.2%)	47 (94.0%)	
**CBL Mutation**			
Present	5 (9.6%)	1 (2.0%)	0.2052
Absent	47 (90.4%)	49 (98.0%)	
**ERRFI1 Mutation**			
Present	0 (0.0%)	0 (0.0%)	1
Absent	52 (100.0%)	50 (100.0%)	
**NF1 Mutation**			
Present	10 (19.2%)	2 (4.0%)	**0.02834**
Absent	42 (80.8%)	48 (96.0%)	
**RASA1 Mutation**			
Present	3 (5.8%)	1 (2.0%)	0.6178
Absent	49 (94.2%)	49 (98.0%)	
**PTPN11 Mutation**			
Present	0 (0.0%)	1 (2.0%)	0.4902
Absent	52 (100.0%)	49 (98.0%)	
**RIT1 Mutation**			
Present	1 (1.9%)	0 (0.0%)	1
Absent	51 (98.1%)	50 (100.0%)	
**ARAF Mutation**			
Present	1 (1.9%)	2 (4.0%)	0.6139
Absent	51 (98.1%)	48 (96.0%)	
**RAF1 Mutation**			
Present	2 (3.8%)	1 (2.0%)	1
Absent	50 (96.2%)	49 (98.0%)	
**RAC1 Mutation**			
Present	0 (0.0%)	0 (0.0%)	1
Absent	52 (100.0%)	50 (100.0%)	

Table S5. Comparison of RTK-RAS pathway alteration frequencies in early-onset Non-Hispanic White (NHW) colorectal cancer patients treated with FOLFOX versus not treated with FOLFOX.

RTK/RAS Pathway			
Gene	Early-Onset NHW Treated with FOLFOX n (%)	Early-Onset NHW Not Treated with FOLFOX n (%)	p-value
**EGFR Mutation**			
Present	5 (1.3%)	5 (1.7%)	0.98
Absent	370 (98.7%)	297 (98.3%)	
**ERBB2 Mutation**			
Present	15 (4.0%)	16 (5.3%)	0.5364
Absent	360 (96.0%)	286 (94.7%)	
**ERBB3 Mutation**			
Present	14 (3.7%)	17 (5.6%)	0.3231
Absent	361 (96.3%)	285 (94.4%)	
**ERBB4 Mutation**			
Present	15 (4.0%)	17 (5.6%)	0.4175
Absent	360 (96.0%)	285 (94.4%)	
**FGFR1 Mutation**			
Present	10 (2.7%)	6 (2.0%)	0.7456
Absent	365 (97.3%)	296 (98.0%)	
**FGFR2 Mutation**			
Present	5 (1.3%)	9 (3.0%)	0.2206
Absent	370 (98.7%)	293 (97.0%)	
**FGFR3 Mutation**			
Present	5 (1.3%)	11 (3.6%)	0.08699
Absent	370 (98.7%)	291 (96.4%)	
**FGFR4 Mutation**			
Present	6 (1.6%)	5 (1.7%)	1
Absent	369 (98.4%)	297 (98.3%)	
**KRAS Mutation**			
Present	163 (43.5%)	124 (41.1%)	0.5811
Absent	212 (56.5%)	178 (58.9%)	
**NRAS Mutation**			
Present	12 (3.2%)	6 (2.0%)	0.4623
Absent	363 (96.8%)	296 (98.0%)	
**HRAS Mutation**			
Present	3 (0.8%)	2 (0.7%)	1
Absent	372 (99.2%)	300 (99.3%)	
**BRAF Mutation**			
Present	27 (7.2%)	24 (7.9%)	0.8262
Absent	348 (92.8%)	278 (92.1%)	
**MAP2K1 Mutation**			
Present	6 (1.6%)	4 (1.3%)	1
Absent	369 (98.4%)	298 (98.7%)	
**MAP2K2 Mutation**			
Present	3 (0.8%)	1 (0.3%)	0.6326
Absent	372 (99.2%)	301 (99.7%)	
**MAPK1 Mutation**			
Present	3 (0.8%)	3 (1.0%)	1
Absent	372 (99.2%)	299 (99.0%)	
**MAPK3 Mutation**			
Present	1 (0.3%)	3 (1.0%)	0.3291
Absent	374 (99.7%)	299 (99.0%)	
**SOS1 Mutation**			
Present	7 (1.9%)	11 (3.6%)	0.2351
Absent	368 (98.1%)	291 (96.4%)	
**MET Mutation**			
Present	4 (1.1%)	10 (3.3%)	0.05575
Absent	371 (98.9%)	292 (96.7%)	
**PDGFRA Mutation**			
Present	14 (3.7%)	16 (5.3%)	0.4263
Absent	361 (96.3%)	286 (94.7%)	
**KIT Mutation**			
Present	8 (2.1%)	13 (4.3%)	0.1625
Absent	367 (97.9%)	289 (95.7%)	
**IGF1R Mutation**			
Present	8 (2.1%)	16 (5.3%)	**0.04502**
Absent	367 (97.9%)	286 (94.7%)	
**RET Mutation**			
Present	8 (2.1%)	11 (3.6%)	0.3433
Absent	367 (97.9%)	291 (96.4%)	
**ROS1 Mutation**			
Present	12 (3.2%)	13 (4.3%)	0.5805
Absent	363 (96.8%)	289 (95.7%)	
**ALK Mutation**			
Present	16 (4.3%)	21 (7.0%)	0.1742
Absent	359 (95.7%)	281 (93.0%)	
**FLT3 Mutation**			
Present	3 (0.8%)	10 (3.3%)	**0.02303**
Absent	372 (99.2%)	292 (96.7%)	
**NTRK1 Mutation**			
Present	10 (2.7%)	9 (3.0%)	0.9909
Absent	365 (97.3%)	293 (97.0%)	
**NTRK2 Mutation**			
Present	5 (1.3%)	8 (2.6%)	0.3379
Absent	370 (98.7%)	294 (97.4%)	
**CBL Mutation**			
Present	4 (1.1%)	4 (1.3%)	1
Absent	371 (98.9%)	298 (98.7%)	
**ERRFI1 Mutation**			
Present	2 (0.5%)	8 (2.6%)	**0.02756**
Absent	373 (99.5%)	294 (97.4%)	
**NF1 Mutation**			
Present	17 (4.5%)	18 (6.0%)	0.5099
Absent	358 (95.5%)	284 (94.0%)	
**RASA1 Mutation**			
Present	9 (2.4%)	14 (4.6%)	0.1667
Absent	366 (97.6%)	288 (95.4%)	
**PTPN11 Mutation**			
Present	5 (1.3%)	5 (1.7%)	0.98
Absent	370 (98.7%)	297 (98.3%)	
**RIT1 Mutation**			
Present	3 (0.8%)	5 (1.7%)	0.4772
Absent	372 (99.2%)	297 (98.3%)	
**ARAF Mutation**			
Present	7 (1.9%)	4 (1.3%)	0.7623
Absent	368 (98.1%)	298 (98.7%)	
**RAF1 Mutation**			
Present	9 (2.4%)	9 (3.0%)	0.8211
Absent	366 (97.6%)	293 (97.0%)	
**RAC1 Mutation**			
Present	1 (0.3%)	3 (1.0%)	0.3291
Absent	374 (99.7%)	299 (99.0%)	

Table S6. Comparison of RTK-RAS pathway alteration frequencies in late-onset Non-Hispanic White (NHW) colorectal cancer patients treated with FOLFOX versus not treated with FOLFOX.

RTK/RAS Pathway			
Gene	Late-Onset NHW Treated with FOLFOX n (%)	Late-Onset NHW Not Treated with FOLFOX n (%)	p-value
**EGFR Mutation**			
Present	22 (2.4%)	24 (3.7%)	0.1823
Absent	897 (97.6%)	629 (96.3%)	
**ERBB2 Mutation**			
Present	40 (4.4%)	43 (6.6%)	0.06636
Absent	879 (95.6%)	610 (93.4%)	
**ERBB3 Mutation**			
Present	35 (3.8%)	47 (7.2%)	**0.004198**
Absent	884 (96.2%)	606 (92.8%)	
**ERBB4 Mutation**			
Present	41 (4.5%)	41 (6.3%)	0.1384
Absent	878 (95.5%)	612 (93.7%)	
**FGFR1 Mutation**			
Present	15 (1.6%)	16 (2.5%)	0.3343
Absent	904 (98.4%)	637 (97.5%)	
**FGFR2 Mutation**			
Present	8 (0.9%)	11 (1.7%)	0.222
Absent	911 (99.1%)	642 (98.3%)	
**FGFR3 Mutation**			
Present	16 (1.7%)	19 (2.9%)	0.1694
Absent	903 (98.3%)	634 (97.1%)	
**FGFR4 Mutation**			
Present	17 (1.8%)	22 (3.4%)	0.08119
Absent	902 (98.2%)	631 (96.6%)	
**KRAS Mutation**			
Present	403 (43.9%)	279 (42.7%)	0.6948
Absent	516 (56.1%)	374 (57.3%)	
**NRAS Mutation**			
Present	36 (3.9%)	19 (2.9%)	0.3512
Absent	883 (96.1%)	634 (97.1%)	
**HRAS Mutation**			
Present	4 (0.4%)	6 (0.9%)	0.3353
Absent	915 (99.6%)	647 (99.1%)	
**BRAF Mutation**			
Present	102 (11.1%)	93 (14.2%)	0.07422
Absent	817 (88.9%)	560 (85.8%)	
**MAP2K1 Mutation**			
Present	12 (1.3%)	10 (1.5%)	0.8749
Absent	907 (98.7%)	643 (98.5%)	
**MAP2K2 Mutation**			
Present	7 (0.8%)	10 (1.5%)	0.2276
Absent	912 (99.2%)	643 (98.5%)	
**MAPK1 Mutation**			
Present	1 (0.1%)	6 (0.9%)	0.02292
Absent	918 (99.9%)	647 (99.1%)	
**MAPK3 Mutation**			
Present	4 (0.4%)	5 (0.8%)	0.5023
Absent	915 (99.6%)	648 (99.2%)	
**SOS1 Mutation**			
Present	8 (0.9%)	15 (2.3%)	0.03501
Absent	911 (99.1%)	638 (97.7%)	
**MET Mutation**			
Present	12 (1.3%)	17 (2.6%)	0.09028
Absent	907 (98.7%)	636 (97.4%)	
**PDGFRA Mutation**			
Present	18 (2.0%)	24 (3.7%)	0.05469
Absent	901 (98.0%)	629 (96.3%)	
**KIT Mutation**			
Present	14 (1.5%)	26 (4.0%)	**0.003883**
Absent	905 (98.5%)	627 (96.0%)	
**IGF1R Mutation**			
Present	28 (3.0%)	29 (4.4%)	0.1867
Absent	891 (97.0%)	624 (95.6%)	
**RET Mutation**			
Present	19 (2.1%)	33 (5.1%)	**0.001813**
Absent	900 (97.9%)	620 (94.9%)	
**ROS1 Mutation**			
Present	31 (3.4%)	30 (4.6%)	0.2702
Absent	888 (96.6%)	623 (95.4%)	
**ALK Mutation**			
Present	29 (3.2%)	39 (6.0%)	**0.009893**
Absent	890 (96.8%)	614 (94.0%)	
**FLT3 Mutation**			
Present	13 (1.4%)	18 (2.8%)	0.08881
Absent	906 (98.6%)	635 (97.2%)	
**NTRK1 Mutation**			
Present	14 (1.5%)	14 (2.1%)	0.4696
Absent	905 (98.5%)	639 (97.9%)	
**NTRK2 Mutation**			
Present	16 (1.7%)	9 (1.4%)	0.7173
Absent	903 (98.3%)	644 (98.6%)	
**CBL Mutation**			
Present	8 (0.9%)	8 (1.2%)	0.6633
Absent	911 (99.1%)	645 (98.8%)	
**ERRFI1 Mutation**			
Present	6 (0.7%)	6 (0.9%)	0.7619
Absent	913 (99.3%)	647 (99.1%)	
**NF1 Mutation**			
Present	46 (5.0%)	44 (6.7%)	0.178
Absent	873 (95.0%)	609 (93.3%)	
**RASA1 Mutation**			
Present	19 (2.1%)	24 (3.7%)	0.07688
Absent	900 (97.9%)	629 (96.3%)	
**PTPN11 Mutation**			
Present	9 (1.0%)	9 (1.4%)	0.6227
Absent	910 (99.0%)	644 (98.6%)	
**RIT1 Mutation**			
Present	6 (0.7%)	5 (0.8%)	1
Absent	913 (99.3%)	648 (99.2%)	
**ARAF Mutation**			
Present	12 (1.3%)	19 (2.9%)	**0.03847**
Absent	907 (98.7%)	634 (97.1%)	
**RAF1 Mutation**			
Present	8 (0.9%)	19 (2.9%)	**0.004111**
Absent	911 (99.1%)	634 (97.1%)	
**RAC1 Mutation**			
Present	0 (0.0%)	3 (0.5%)	0.07148
Absent	919 (100.0%)	650 (99.5%)	

Table S7. Comparison of RTK-RAS pathway alteration frequencies between early-onset and late-onset Non-Hispanic White (NHW) colorectal cancer patients treated with FOLFOX.

RTK/RAS Pathway			
**Gene**	**Early-Onset NHW Treated with FOLFOX n (%)**	**Late-Onset NHW Treated with FOLFOX n (%)**	**p-value**
**EGFR Mutation**			
Present	5 (1.3%)	22 (2.4%)	0.319
Absent	370 (98.7%)	897 (97.6%)	
**ERBB2 Mutation**			
Present	15 (4.0%)	40 (4.4%)	0.8939
Absent	360 (96.0%)	879 (95.6%)	
**ERBB3 Mutation**			
Present	14 (3.7%)	35 (3.8%)	1
Absent	361 (96.3%)	884 (96.2%)	
**ERBB4 Mutation**			
Present	15 (4.0%)	41 (4.5%)	0.8263
Absent	360 (96.0%)	878 (95.5%)	
**FGFR1 Mutation**			
Present	10 (2.7%)	15 (1.6%)	0.3154
Absent	365 (97.3%)	904 (98.4%)	
**FGFR2 Mutation**			
Present	5 (1.3%)	8 (0.9%)	0.6526
Absent	370 (98.7%)	911 (99.1%)	
**FGFR3 Mutation**			
Present	5 (1.3%)	16 (1.7%)	0.7763
Absent	370 (98.7%)	903 (98.3%)	
**FGFR4 Mutation**			
Present	6 (1.6%)	17 (1.8%)	0.9389
Absent	369 (98.4%)	902 (98.2%)	
**KRAS Mutation**			
Present	163 (43.5%)	403 (43.9%)	0.9482
Absent	212 (56.5%)	516 (56.1%)	
**NRAS Mutation**			
Present	12 (3.2%)	36 (3.9%)	0.6475
Absent	363 (96.8%)	883 (96.1%)	
**HRAS Mutation**			
Present	3 (0.8%)	4 (0.4%)	1
Absent	372 (99.2%)	915 (99.6%)	
**BRAF Mutation**			
Present	27 (7.2%)	102 (11.1%)	**0.04321**
Absent	348 (92.8%)	817 (88.9%)	
**MAP2K1 Mutation**			
Present	6 (1.6%)	12 (1.3%)	0.882
Absent	369 (98.4%)	907 (98.7%)	
**MAP2K2 Mutation**			
Present	3 (0.8%)	7 (0.8%)	1
Absent	372 (99.2%)	912 (99.2%)	
**MAPK1 Mutation**			
Present	3 (0.8%)	1 (0.1%)	0.07588
Absent	372 (99.2%)	918 (99.9%)	
**MAPK3 Mutation**			
Present	1 (0.3%)	4 (0.4%)	1
Absent	374 (99.7%)	915 (99.6%)	
**SOS1 Mutation**			
Present	7 (1.9%)	8 (0.9%)	0.2178
Absent	368 (98.1%)	911 (99.1%)	
**MET Mutation**			
Present	4 (1.1%)	12 (1.3%)	1
Absent	371 (98.9%)	907 (98.7%)	
**PDGFRA Mutation**			
Present	14 (3.7%)	18 (2.0%)	0.09539
Absent	361 (96.3%)	901 (98.0%)	
**KIT Mutation**			
Present	8 (2.1%)	14 (1.5%)	0.5941
Absent	367 (97.9%)	905 (98.5%)	
**IGF1R Mutation**			
Present	8 (2.1%)	28 (3.0%)	0.4714
Absent	367 (97.9%)	891 (97.0%)	
**RET Mutation**			
Present	8 (2.1%)	19 (2.1%)	1
Absent	367 (97.9%)	900 (97.9%)	
**ROS1 Mutation**			
Present	12 (3.2%)	31 (3.4%)	1
Absent	363 (96.8%)	888 (96.6%)	
**ALK Mutation**			
Present	16 (4.3%)	29 (3.2%)	0.4108
Absent	359 (95.7%)	890 (96.8%)	
**FLT3 Mutation**			
Present	3 (0.8%)	13 (1.4%)	0.5793
Absent	372 (99.2%)	906 (98.6%)	
**NTRK1 Mutation**			
Present	10 (2.7%)	14 (1.5%)	0.2478
Absent	365 (97.3%)	905 (98.5%)	
**NTRK2 Mutation**			
Present	5 (1.3%)	16 (1.7%)	0.7763
Absent	370 (98.7%)	903 (98.3%)	
**CBL Mutation**			
Present	4 (1.1%)	8 (0.9%)	0.7529
Absent	371 (98.9%)	911 (99.1%)	
**ERRFI1 Mutation**			
Present	2 (0.5%)	6 (0.7%)	1
Absent	373 (99.5%)	913 (99.3%)	
**NF1 Mutation**			
Present	17 (4.5%)	46 (5.0%)	0.8293
Absent	358 (95.5%)	873 (95.0%)	
**RASA1 Mutation**			
Present	9 (2.4%)	19 (2.1%)	0.871
Absent	366 (97.6%)	900 (97.9%)	
**PTPN11 Mutation**			
Present	5 (1.3%)	9 (1.0%)	0.7931
Absent	370 (98.7%)	910 (99.0%)	
**RIT1 Mutation**			
Present	3 (0.8%)	6 (0.7%)	0.7238
Absent	372 (99.2%)	913 (99.3%)	
**ARAF Mutation**			
Present	7 (1.9%)	12 (1.3%)	0.6063
Absent	368 (98.1%)	907 (98.7%)	
**RAF1 Mutation**			
Present	9 (2.4%)	8 (0.9%)	0.05447
Absent	366 (97.6%)	911 (99.1%)	
**RAC1 Mutation**			
Present	1 (0.3%)	0 (0.0%)	0.2898
Absent	374 (99.7%)	919 (100.0%)	

Table S8. Comparison of RTK-RAS pathway alteration frequencies between early-onset and late-onset Non-Hispanic White (NHW) colorectal cancer patients not treated with FOLFOX.

RTK/RAS Pathway			
Gene	Early-Onset NHW Not Treated with FOLFOX n (%)	Late-Onset NHW Not Treated with FOLFOX n (%)	p-value
**EGFR Mutation**			
Present	5 (1.7%)	24 (3.7%)	0.1366
Absent	297 (98.3%)	629 (96.3%)	
**ERBB2 Mutation**			
Present	16 (5.3%)	43 (6.6%)	0.5329
Absent	286 (94.7%)	610 (93.4%)	
**ERBB3 Mutation**			
Present	17 (5.6%)	47 (7.2%)	0.4459
Absent	285 (94.4%)	606 (92.8%)	
**ERBB4 Mutation**			
Present	17 (5.6%)	41 (6.3%)	0.8063
Absent	285 (94.4%)	612 (93.7%)	
**FGFR1 Mutation**			
Present	6 (2.0%)	16 (2.5%)	0.8321
Absent	296 (98.0%)	637 (97.5%)	
**FGFR2 Mutation**			
Present	9 (3.0%)	11 (1.7%)	0.2904
Absent	293 (97.0%)	642 (98.3%)	
**FGFR3 Mutation**			
Present	11 (3.6%)	19 (2.9%)	0.6861
Absent	291 (96.4%)	634 (97.1%)	
**FGFR4 Mutation**			
Present	5 (1.7%)	22 (3.4%)	0.2021
Absent	297 (98.3%)	631 (96.6%)	
**KRAS Mutation**			
Present	124 (41.1%)	279 (42.7%)	0.6786
Absent	178 (58.9%)	374 (57.3%)	
**NRAS Mutation**			
Present	6 (2.0%)	19 (2.9%)	0.5401
Absent	296 (98.0%)	634 (97.1%)	
**HRAS Mutation**			
Present	2 (0.7%)	6 (0.9%)	1
Absent	300 (99.3%)	647 (99.1%)	
**BRAF Mutation**			
Present	24 (7.9%)	93 (14.2%)	**0.007983**
Absent	278 (92.1%)	560 (85.8%)	
**MAP2K1 Mutation**			
Present	4 (1.3%)	10 (1.5%)	1
Absent	298 (98.7%)	643 (98.5%)	
**MAP2K2 Mutation**			
Present	1 (0.3%)	10 (1.5%)	0.1885
Absent	301 (99.7%)	643 (98.5%)	
**MAPK1 Mutation**			
Present	3 (1.0%)	6 (0.9%)	1
Absent	299 (99.0%)	647 (99.1%)	
**MAPK3 Mutation**			
Present	3 (1.0%)	5 (0.8%)	0.713
Absent	299 (99.0%)	648 (99.2%)	
**SOS1 Mutation**			
Present	11 (3.6%)	15 (2.3%)	0.33
Absent	291 (96.4%)	638 (97.7%)	
**MET Mutation**			
Present	10 (3.3%)	17 (2.6%)	0.6864
Absent	292 (96.7%)	636 (97.4%)	
**PDGFRA Mutation**			
Present	16 (5.3%)	24 (3.7%)	0.322
Absent	286 (94.7%)	629 (96.3%)	
**KIT Mutation**			
Present	13 (4.3%)	26 (4.0%)	0.9532
Absent	289 (95.7%)	627 (96.0%)	
**IGF1R Mutation**			
Present	16 (5.3%)	29 (4.4%)	0.6767
Absent	286 (94.7%)	624 (95.6%)	
**RET Mutation**			
Present	11 (3.6%)	33 (5.1%)	0.4229
Absent	291 (96.4%)	620 (94.9%)	
**ROS1 Mutation**			
Present	13 (4.3%)	30 (4.6%)	0.9738
Absent	289 (95.7%)	623 (95.4%)	
**ALK Mutation**			
Present	21 (7.0%)	39 (6.0%)	0.6616
Absent	281 (93.0%)	614 (94.0%)	
**FLT3 Mutation**			
Present	10 (3.3%)	18 (2.8%)	0.79
Absent	292 (96.7%)	635 (97.2%)	
**NTRK1 Mutation**			
Present	9 (3.0%)	14 (2.1%)	0.5777
Absent	293 (97.0%)	639 (97.9%)	
**NTRK2 Mutation**			
Present	8 (2.6%)	9 (1.4%)	0.2636
Absent	294 (97.4%)	644 (98.6%)	
**CBL Mutation**			
Present	4 (1.3%)	8 (1.2%)	1
Absent	298 (98.7%)	645 (98.8%)	
**ERRFI1 Mutation**			
Present	8 (2.6%)	6 (0.9%)	0.07521
Absent	294 (97.4%)	647 (99.1%)	
**NF1 Mutation**			
Present	18 (6.0%)	44 (6.7%)	0.7547
Absent	284 (94.0%)	609 (93.3%)	
**RASA1 Mutation**			
Present	14 (4.6%)	24 (3.7%)	0.5975
Absent	288 (95.4%)	629 (96.3%)	
**PTPN11 Mutation**			
Present	5 (1.7%)	9 (1.4%)	0.9664
Absent	297 (98.3%)	644 (98.6%)	
**RIT1 Mutation**			
Present	5 (1.7%)	5 (0.8%)	0.3605
Absent	297 (98.3%)	648 (99.2%)	
**ARAF Mutation**			
Present	4 (1.3%)	19 (2.9%)	0.1748
Absent	298 (98.7%)	634 (97.1%)	
**RAF1 Mutation**			
Present	9 (3.0%)	19 (2.9%)	1
Absent	293 (97.0%)	634 (97.1%)	
**RAC1 Mutation**			
Present	3 (1.0%)	3 (0.5%)	0.3873
Absent	299 (99.0%)	650 (99.5%)	

Table S9. Comparison of RTK-RAS pathway alteration frequencies between early-onset Hispanic/Latino (H/L) and early-onset Non-Hispanic White (NHW) colorectal cancer patients treated with FOLFOX.

RTK/RAS Pathway			
Gene	Early-Onset Hispanic/Latino Treated with FOLFOX n (%)	Early-Onset NHW Treated with FOLFOX n (%)	p-value
**EGFR Mutation**			
Present	1 (1.4%)	5 (1.3%)	1
Absent	72 (98.6%)	370 (98.7%)	
**ERBB2 Mutation**			
Present	2 (2.7%)	15 (4.0%)	1
Absent	71 (97.3%)	360 (96.0%)	
**ERBB3 Mutation**			
Present	4 (5.5%)	14 (3.7%)	0.5121
Absent	69 (94.5%)	361 (96.3%)	
**ERBB4 Mutation**			
Present	3 (4.1%)	15 (4.0%)	1
Absent	70 (95.9%)	360 (96.0%)	
**FGFR1 Mutation**			
Present	0 (0.0%)	10 (2.7%)	0.3783
Absent	73 (100.0%)	365 (97.3%)	
**FGFR2 Mutation**			
Present	0 (0.0%)	5 (1.3%)	1
Absent	73 (100.0%)	370 (98.7%)	
**FGFR3 Mutation**			
Present	1 (1.4%)	5 (1.3%)	1
Absent	72 (98.6%)	370 (98.7%)	
**FGFR4 Mutation**			
Present	1 (1.4%)	6 (1.6%)	1
Absent	72 (98.6%)	369 (98.4%)	
**KRAS Mutation**			
Present	30 (41.1%)	163 (43.5%)	0.8064
Absent	43 (58.9%)	212 (56.5%)	
**NRAS Mutation**			
Present	2 (2.7%)	12 (3.2%)	1
Absent	71 (97.3%)	363 (96.8%)	
**HRAS Mutation**			
Present	1 (1.4%)	3 (0.8%)	0.5104
Absent	72 (98.6%)	372 (99.2%)	
**BRAF Mutation**			
Present	3 (4.1%)	27 (7.2%)	0.4472
Absent	70 (95.9%)	348 (92.8%)	
**MAP2K1 Mutation**			
Present	2 (2.7%)	6 (1.6%)	0.6222
Absent	71 (97.3%)	369 (98.4%)	
**MAP2K2 Mutation**			
Present	1 (1.4%)	3 (0.8%)	0.5104
Absent	72 (98.6%)	372 (99.2%)	
**MAPK1 Mutation**			
Present	0 (0.0%)	3 (0.8%)	1
Absent	73 (100.0%)	372 (99.2%)	
**MAPK3 Mutation**			
Present	1 (1.4%)	1 (0.3%)	0.2996
Absent	72 (98.6%)	374 (99.7%)	
**SOS1 Mutation**			
Present	0 (0.0%)	7 (1.9%)	0.6049
Absent	73 (100.0%)	368 (98.1%)	
**MET Mutation**			
Present	1 (1.4%)	4 (1.1%)	0.5909
Absent	72 (98.6%)	371 (98.9%)	
**PDGFRA Mutation**			
Present	2 (2.7%)	14 (3.7%)	1
Absent	71 (97.3%)	361 (96.3%)	
**KIT Mutation**			
Present	1 (1.4%)	8 (2.1%)	1
Absent	72 (98.6%)	367 (97.9%)	
**IGF1R Mutation**			
Present	1 (1.4%)	8 (2.1%)	1
Absent	72 (98.6%)	367 (97.9%)	
**RET Mutation**			
Present	1 (1.4%)	8 (2.1%)	1
Absent	72 (98.6%)	367 (97.9%)	
**ROS1 Mutation**			
Present	2 (2.7%)	12 (3.2%)	1
Absent	71 (97.3%)	363 (96.8%)	
**ALK Mutation**			
Present	2 (2.7%)	16 (4.3%)	0.7499
Absent	71 (97.3%)	359 (95.7%)	
**FLT3 Mutation**			
Present	1 (1.4%)	3 (0.8%)	0.5104
Absent	72 (98.6%)	372 (99.2%)	
**NTRK1 Mutation**			
Present	1 (1.4%)	10 (2.7%)	1
Absent	72 (98.6%)	365 (97.3%)	
**NTRK2 Mutation**			
Present	1 (1.4%)	5 (1.3%)	1
Absent	72 (98.6%)	370 (98.7%)	
**CBL Mutation**			
Present	2 (2.7%)	4 (1.1%)	0.2538
Absent	71 (97.3%)	371 (98.9%)	
**ERRFI1 Mutation**			
Present	0 (0.0%)	2 (0.5%)	1
Absent	73 (100.0%)	373 (99.5%)	
**NF1 Mutation**			
Present	3 (4.1%)	17 (4.5%)	1
Absent	70 (95.9%)	358 (95.5%)	
**RASA1 Mutation**			
Present	1 (1.4%)	9 (2.4%)	1
Absent	72 (98.6%)	366 (97.6%)	
**PTPN11 Mutation**			
Present	0 (0.0%)	5 (1.3%)	1
Absent	73 (100.0%)	370 (98.7%)	
**RIT1 Mutation**			
Present	1 (1.4%)	3 (0.8%)	0.5104
Absent	72 (98.6%)	372 (99.2%)	
**ARAF Mutation**			
Present	0 (0.0%)	7 (1.9%)	0.6049
Absent	73 (100.0%)	368 (98.1%)	
**RAF1 Mutation**			
Present	1 (1.4%)	9 (2.4%)	1
Absent	72 (98.6%)	366 (97.6%)	
**RAC1 Mutation**			
Present	0 (0.0%)	1 (0.3%)	1
Absent	73 (100.0%)	374 (99.7%)	

Table S10. Comparison of RTK-RAS pathway alteration frequencies between early-onset Hispanic/Latino (H/L) and early-onset Non-Hispanic White (NHW) colorectal cancer patients not treated with FOLFOX.

RTK/RAS Pathway			
Gene	Early-Onset Hispanic/Latino Not Treated with FOLFOX n (%)	Early-Onset NHW Not Treated with FOLFOX n (%)	p-value
**EGFR Mutation**			
Present	3 (5.8%)	5 (1.7%)	0.09778
Absent	49 (94.2%)	297 (98.3%)	
**ERBB2 Mutation**			
Present	8 (15.4%)	16 (5.3%)	**0.01761**
Absent	44 (84.6%)	286 (94.7%)	
**ERBB3 Mutation**			
Present	3 (5.8%)	17 (5.6%)	1
Absent	49 (94.2%)	285 (94.4%)	
**ERBB4 Mutation**			
Present	7 (13.5%)	17 (5.6%)	0.07565
Absent	45 (86.5%)	285 (94.4%)	
**FGFR1 Mutation**			
Present	2 (3.8%)	6 (2.0%)	0.3331
Absent	50 (96.2%)	296 (98.0%)	
**FGFR2 Mutation**			
Present	4 (7.7%)	9 (3.0%)	0.1073
Absent	48 (92.3%)	293 (97.0%)	
**FGFR3 Mutation**			
Present	2 (3.8%)	11 (3.6%)	1
Absent	50 (96.2%)	291 (96.4%)	
**FGFR4 Mutation**			
Present	1 (1.9%)	5 (1.7%)	1
Absent	51 (98.1%)	297 (98.3%)	
**KRAS Mutation**			
Present	18 (34.6%)	124 (41.1%)	0.47
Absent	34 (65.4%)	178 (58.9%)	
**NRAS Mutation**			
Present	3 (5.8%)	6 (2.0%)	0.1318
Absent	49 (94.2%)	296 (98.0%)	
**HRAS Mutation**			
Present	1 (1.9%)	2 (0.7%)	0.38
Absent	51 (98.1%)	300 (99.3%)	
**BRAF Mutation**			
Present	4 (7.7%)	24 (7.9%)	1
Absent	48 (92.3%)	278 (92.1%)	
**MAP2K1 Mutation**			
Present	3 (5.8%)	4 (1.3%)	0.06807
Absent	49 (94.2%)	298 (98.7%)	
**MAP2K2 Mutation**			
Present	1 (1.9%)	1 (0.3%)	0.2726
Absent	51 (98.1%)	301 (99.7%)	
**MAPK1 Mutation**			
Present	2 (3.8%)	3 (1.0%)	0.158
Absent	50 (96.2%)	299 (99.0%)	
**MAPK3 Mutation**			
Present	3 (5.8%)	3 (1.0%)	**0.04335**
Absent	49 (94.2%)	299 (99.0%)	
**SOS1 Mutation**			
Present	1 (1.9%)	11 (3.6%)	1
Absent	51 (98.1%)	291 (96.4%)	
**MET Mutation**			
Present	0 (0.0%)	10 (3.3%)	0.369
Absent	52 (100.0%)	292 (96.7%)	
**PDGFRA Mutation**			
Present	5 (9.6%)	16 (5.3%)	0.3684
Absent	47 (90.4%)	286 (94.7%)	
**KIT Mutation**			
Present	3 (5.8%)	13 (4.3%)	0.7147
Absent	49 (94.2%)	289 (95.7%)	
**IGF1R Mutation**			
Present	4 (7.7%)	16 (5.3%)	0.5127
Absent	48 (92.3%)	286 (94.7%)	
**RET Mutation**			
Present	1 (1.9%)	11 (3.6%)	1
Absent	51 (98.1%)	291 (96.4%)	
**ROS1 Mutation**			
Present	4 (7.7%)	13 (4.3%)	0.2914
Absent	48 (92.3%)	289 (95.7%)	
**ALK Mutation**			
Present	2 (3.8%)	21 (7.0%)	0.5507
Absent	50 (96.2%)	281 (93.0%)	
**FLT3 Mutation**			
Present	4 (7.7%)	10 (3.3%)	0.1342
Absent	48 (92.3%)	292 (96.7%)	
**NTRK1 Mutation**			
Present	2 (3.8%)	9 (3.0%)	0.668
Absent	50 (96.2%)	293 (97.0%)	
**NTRK2 Mutation**			
Present	2 (3.8%)	8 (2.6%)	0.6456
Absent	50 (96.2%)	294 (97.4%)	
**CBL Mutation**			
Present	5 (9.6%)	4 (1.3%)	**0.004522**
Absent	47 (90.4%)	298 (98.7%)	
**ERRFI1 Mutation**			
Present	0 (0.0%)	8 (2.6%)	0.6098
Absent	52 (100.0%)	294 (97.4%)	
**NF1 Mutation**			
Present	10 (19.2%)	18 (6.0%)	**0.002728**
Absent	42 (80.8%)	284 (94.0%)	
**RASA1 Mutation**			
Present	3 (5.8%)	14 (4.6%)	0.7245
Absent	49 (94.2%)	288 (95.4%)	
**PTPN11 Mutation**			
Present	0 (0.0%)	5 (1.7%)	1
Absent	52 (100.0%)	297 (98.3%)	
**RIT1 Mutation**			
Present	1 (1.9%)	5 (1.7%)	1
Absent	51 (98.1%)	297 (98.3%)	
**ARAF Mutation**			
Present	1 (1.9%)	4 (1.3%)	0.5503
Absent	51 (98.1%)	298 (98.7%)	
**RAF1 Mutation**			
Present	2 (3.8%)	9 (3.0%)	0.668
Absent	50 (96.2%)	293 (97.0%)	
**RAC1 Mutation**			
Present	0 (0.0%)	3 (1.0%)	1
Absent	52 (100.0%)	299 (99.0%)	

Table S11. Comparison of RTK-RAS pathway alteration frequencies between late-onset Hispanic/Latino (H/L) and late-onset Non-Hispanic White (NHW) colorectal cancer patients treated with FOLFOX.

RTK/RAS Pathway			
**Gene**	**Late-Onset Hispanic/Latino Treated with FOLFOX n (%)**	**Late-Onset NHW Treated with FOLFOX n (%)**	**p-value**
**EGFR Mutation**			
Present	1 (1.1%)	22 (2.4%)	0.7141
Absent	90 (98.9%)	897 (97.6%)	
**ERBB2 Mutation**			
Present	2 (2.2%)	40 (4.4%)	0.5774
Absent	89 (97.8%)	879 (95.6%)	
**ERBB3 Mutation**			
Present	1 (1.1%)	35 (3.8%)	0.2443
Absent	90 (98.9%)	884 (96.2%)	
**ERBB4 Mutation**			
Present	3 (3.3%)	41 (4.5%)	0.7905
Absent	88 (96.7%)	878 (95.5%)	
**FGFR1 Mutation**			
Present	0 (0.0%)	15 (1.6%)	0.386
Absent	91 (100.0%)	904 (98.4%)	
**FGFR2 Mutation**			
Present	0 (0.0%)	8 (0.9%)	1
Absent	91 (100.0%)	911 (99.1%)	
**FGFR3 Mutation**			
Present	3 (3.3%)	16 (1.7%)	0.241
Absent	88 (96.7%)	903 (98.3%)	
**FGFR4 Mutation**			
Present	1 (1.1%)	17 (1.8%)	1
Absent	90 (98.9%)	902 (98.2%)	
**KRAS Mutation**			
Present	39 (42.9%)	403 (43.9%)	0.9428
Absent	52 (57.1%)	516 (56.1%)	
**NRAS Mutation**			
Present	6 (6.6%)	36 (3.9%)	0.3449
Absent	85 (93.4%)	883 (96.1%)	
**HRAS Mutation**			
Present	0 (0.0%)	4 (0.4%)	1
Absent	91 (100.0%)	915 (99.6%)	
**BRAF Mutation**			
Present	15 (16.5%)	102 (11.1%)	0.1741
Absent	76 (83.5%)	817 (88.9%)	
**MAP2K1 Mutation**			
Present	0 (0.0%)	12 (1.3%)	0.6152
Absent	91 (100.0%)	907 (98.7%)	
**MAP2K2 Mutation**			
Present	2 (2.2%)	7 (0.8%)	0.191
Absent	89 (97.8%)	912 (99.2%)	
**MAPK1 Mutation**			
Present	0 (0.0%)	1 (0.1%)	1
Absent	91 (100.0%)	918 (99.9%)	
**MAPK3 Mutation**			
Present	1 (1.1%)	4 (0.4%)	0.3769
Absent	90 (98.9%)	915 (99.6%)	
**SOS1 Mutation**			
Present	3 (3.3%)	8 (0.9%)	0.06862
Absent	88 (96.7%)	911 (99.1%)	
**MET Mutation**			
Present	4 (4.4%)	12 (1.3%)	**0.04833**
Absent	87 (95.6%)	907 (98.7%)	
**PDGFRA Mutation**			
Present	0 (0.0%)	18 (2.0%)	0.3962
Absent	91 (100.0%)	901 (98.0%)	
**KIT Mutation**			
Present	1 (1.1%)	14 (1.5%)	1
Absent	90 (98.9%)	905 (98.5%)	
**IGF1R Mutation**			
Present	1 (1.1%)	28 (3.0%)	0.507
Absent	90 (98.9%)	891 (97.0%)	
**RET Mutation**			
Present	3 (3.3%)	19 (2.1%)	0.4401
Absent	88 (96.7%)	900 (97.9%)	
**ROS1 Mutation**			
Present	6 (6.6%)	31 (3.4%)	0.1359
Absent	85 (93.4%)	888 (96.6%)	
**ALK Mutation**			
Present	4 (4.4%)	29 (3.2%)	0.5303
Absent	87 (95.6%)	890 (96.8%)	
**FLT3 Mutation**			
Present	2 (2.2%)	13 (1.4%)	0.6379
Absent	89 (97.8%)	906 (98.6%)	
**NTRK1 Mutation**			
Present	2 (2.2%)	14 (1.5%)	0.6487
Absent	89 (97.8%)	905 (98.5%)	
**NTRK2 Mutation**			
Present	0 (0.0%)	16 (1.7%)	0.3866
Absent	91 (100.0%)	903 (98.3%)	
**CBL Mutation**			
Present	0 (0.0%)	8 (0.9%)	1
Absent	91 (100.0%)	911 (99.1%)	
**ERRFI1 Mutation**			
Present	0 (0.0%)	6 (0.7%)	1
Absent	91 (100.0%)	913 (99.3%)	
**NF1 Mutation**			
Present	2 (2.2%)	46 (5.0%)	0.3063
Absent	89 (97.8%)	873 (95.0%)	
**RASA1 Mutation**			
Present	4 (4.4%)	19 (2.1%)	0.1454
Absent	87 (95.6%)	900 (97.9%)	
**PTPN11 Mutation**			
Present	2 (2.2%)	9 (1.0%)	0.2603
Absent	89 (97.8%)	910 (99.0%)	
**RIT1 Mutation**			
Present	1 (1.1%)	6 (0.7%)	0.4847
Absent	90 (98.9%)	913 (99.3%)	
**ARAF Mutation**			
Present	3 (3.3%)	12 (1.3%)	0.1459
Absent	88 (96.7%)	907 (98.7%)	
**RAF1 Mutation**			
Present	2 (2.2%)	8 (0.9%)	0.2255
Absent	89 (97.8%)	911 (99.1%)	
**RAC1 Mutation**			
Present	0 (0.0%)	0 (0.0%)	1
Absent	91 (100.0%)	919 (100.0%)	

Table S12. Comparison of RTK-RAS pathway alteration frequencies between late-onset Hispanic/Latino (H/L) and late-onset Non-Hispanic White (NHW) colorectal cancer patients not treated with FOLFOX.

RTK/RAS Pathway			
Gene	Late-Onset Hispanic/Latino Not Treated with FOLFOX n (%)	Late-Onset NHW Not Treated with FOLFOX n (%)	p-value
**EGFR Mutation**			
Present	3 (6.0%)	24 (3.7%)	0.4311
Absent	47 (94.0%)	629 (96.3%)	
**ERBB2 Mutation**			
Present	5 (10.0%)	43 (6.6%)	0.5275
Absent	45 (90.0%)	610 (93.4%)	
**ERBB3 Mutation**			
Present	3 (6.0%)	47 (7.2%)	1
Absent	47 (94.0%)	606 (92.8%)	
**ERBB4 Mutation**			
Present	3 (6.0%)	41 (6.3%)	1
Absent	47 (94.0%)	612 (93.7%)	
**FGFR1 Mutation**			
Present	0 (0.0%)	16 (2.5%)	0.62
Absent	50 (100.0%)	637 (97.5%)	
**FGFR2 Mutation**			
Present	1 (2.0%)	11 (1.7%)	0.5904
Absent	49 (98.0%)	642 (98.3%)	
**FGFR3 Mutation**			
Present	2 (4.0%)	19 (2.9%)	0.6558
Absent	48 (96.0%)	634 (97.1%)	
**FGFR4 Mutation**			
Present	1 (2.0%)	22 (3.4%)	1
Absent	49 (98.0%)	631 (96.6%)	
**KRAS Mutation**			
Present	20 (40.0%)	279 (42.7%)	0.8202
Absent	30 (60.0%)	374 (57.3%)	
**NRAS Mutation**			
Present	3 (6.0%)	19 (2.9%)	0.2011
Absent	47 (94.0%)	634 (97.1%)	
**HRAS Mutation**			
Present	1 (2.0%)	6 (0.9%)	0.4047
Absent	49 (98.0%)	647 (99.1%)	
**BRAF Mutation**			
Present	8 (16.0%)	93 (14.2%)	0.6786
Absent	42 (84.0%)	560 (85.8%)	
**MAP2K1 Mutation**			
Present	0 (0.0%)	10 (1.5%)	1
Absent	50 (100.0%)	643 (98.5%)	
**MAP2K2 Mutation**			
Present	1 (2.0%)	10 (1.5%)	0.5585
Absent	49 (98.0%)	643 (98.5%)	
**MAPK1 Mutation**			
Present	0 (0.0%)	6 (0.9%)	1
Absent	50 (100.0%)	647 (99.1%)	
**MAPK3 Mutation**			
Present	0 (0.0%)	5 (0.8%)	1
Absent	50 (100.0%)	648 (99.2%)	
**SOS1 Mutation**			
Present	0 (0.0%)	15 (2.3%)	0.6164
Absent	50 (100.0%)	638 (97.7%)	
**MET Mutation**			
Present	2 (4.0%)	17 (2.6%)	0.6387
Absent	48 (96.0%)	636 (97.4%)	
**PDGFRA Mutation**			
Present	0 (0.0%)	24 (3.7%)	0.4049
Absent	50 (100.0%)	629 (96.3%)	
**KIT Mutation**			
Present	0 (0.0%)	26 (4.0%)	0.2473
Absent	50 (100.0%)	627 (96.0%)	
**IGF1R Mutation**			
Present	3 (6.0%)	29 (4.4%)	0.4907
Absent	47 (94.0%)	624 (95.6%)	
**RET Mutation**			
Present	2 (4.0%)	33 (5.1%)	1
Absent	48 (96.0%)	620 (94.9%)	
**ROS1 Mutation**			
Present	2 (4.0%)	30 (4.6%)	1
Absent	48 (96.0%)	623 (95.4%)	
**ALK Mutation**			
Present	2 (4.0%)	39 (6.0%)	0.7604
Absent	48 (96.0%)	614 (94.0%)	
**FLT3 Mutation**			
Present	3 (6.0%)	18 (2.8%)	0.1824
Absent	47 (94.0%)	635 (97.2%)	
**NTRK1 Mutation**			
Present	2 (4.0%)	14 (2.1%)	0.3169
Absent	48 (96.0%)	639 (97.9%)	
**NTRK2 Mutation**			
Present	3 (6.0%)	9 (1.4%)	**0.0472**
Absent	47 (94.0%)	644 (98.6%)	
**CBL Mutation**			
Present	1 (2.0%)	8 (1.2%)	0.4873
Absent	49 (98.0%)	645 (98.8%)	
**ERRFI1 Mutation**			
Present	0 (0.0%)	6 (0.9%)	1
Absent	50 (100.0%)	647 (99.1%)	
**NF1 Mutation**			
Present	2 (4.0%)	44 (6.7%)	0.7642
Absent	48 (96.0%)	609 (93.3%)	
**RASA1 Mutation**			
Present	1 (2.0%)	24 (3.7%)	1
Absent	49 (98.0%)	629 (96.3%)	
**PTPN11 Mutation**			
Present	1 (2.0%)	9 (1.4%)	0.5242
Absent	49 (98.0%)	644 (98.6%)	
**RIT1 Mutation**			
Present	0 (0.0%)	5 (0.8%)	1
Absent	50 (100.0%)	648 (99.2%)	
**ARAF Mutation**			
Present	2 (4.0%)	19 (2.9%)	0.6558
Absent	48 (96.0%)	634 (97.1%)	
**RAF1 Mutation**			
Present	1 (2.0%)	19 (2.9%)	1
Absent	49 (98.0%)	634 (97.1%)	
**RAC1 Mutation**			
Present	0 (0.0%)	3 (0.5%)	1
Absent	50 (100.0%)	650 (99.5%)	

Table S13. Spectrum of RTK-RAS pathway variant classes across colorectal cancer subgroups defined by ancestry, age at diagnosis, and FOLFOX exposure. This table summarizes the relative contribution of distinct somatic variant classes observed in selected RTK-RAS pathway genes, stratified by Hispanic/Latino (H/L) versus non-Hispanic White (NHW) ancestry, early-onset (EOCRC) versus late-onset (LOCRC) disease, and receipt of FOLFOX chemotherapy. Variant categories include truncating, non-truncating, splice-associated, and translation-initiation-related alterations, encompassing both insertion/deletion and single-nucleotide change events. Values are expressed as percentages representing the distribution of each variant class among all detected alterations for a given gene within each subgroup. This stratified presentation enables comparison of mutation pattern heterogeneity across demographic and treatment-defined CRC populations and provides insight into potential differences in underlying mutational processes.

	Hispanic/Latino Samples				Non-Hispanic White Samples			
	Early-Onset		Late-Onset		Early-Onset		Late-Onset	
	Treated with FOLFOX	Not Treated with FOLFOX	Treated with FOLFOX	Not Treated with FOLFOX	Treated with FOLFOX	Not Treated with FOLFOX	Treated with FOLFOX	Not Treated with FOLFOX
**ALK**								
Frame Shift Deletion	0%	0%	0%	50%	10%	15%	7%	14%
Frame Shift Insertion	0%	0%	0%	0%	0%	0%	10%	4%
Missense Mutation	100%	100%	100%	50%	81%	79%	83%	76%
Nonsense Mutation	0%	0%	0%	0%	10%	6%	0%	4%
Splice Site	0%	0%	0%	0%	0%	0%	0%	2%
**ARAF**								
Frame Shift Deletion	0%	100%	67%	50%	38%	25%	42%	25%
Missense Mutation	0%	0%	33%	50%	63%	75%	58%	70%
Splice Site	0%	0%	0%	0%	0%	0%	0%	5%
**BRAF**								
Frame Shift Deletion	0%	0%	0%	0%	0%	4%	4%	1%
Frame Shift Insertion	0%	0%	0%	0%	0%	0%	1%	4%
In Frame Deletion	0%	0%	0%	0%	0%	4%	0%	0%
Missense Mutation	100%	100%	100%	100%	100%	82%	95%	95%
Nonsense Mutation	0%	0%	0%	0%	0%	7%	0%	0%
Splice Site	0%	0%	0%	0%	0%	4%	0%	0%
**CBL**								
Frame Shift Deletion	0%	0%	0%	0%	0%	0%	11%	0%
Frame Shift Insertion	0%	0%	0%	0%	0%	25%	11%	9%
In Frame Insertion	0%	0%	0%	0%	0%	0%	0%	9%
Missense Mutation	100%	100%	0%	100%	75%	75%	67%	82%
Nonsense Mutation	0%	0%	0%	0%	25%	0%	11%	0%
**EGFR**								
Frame Shift Deletion	0%	33%	0%	0%	0%	0%	0%	0%
In Frame Deletion	0%	0%	0%	0%	0%	0%	8%	0%
In Frame Insertion	0%	0%	0%	0%	0%	0%	4%	0%
Missense Mutation	100%	33%	100%	100%	100%	100%	77%	96%
Nonsense Mutation	0%	0%	0%	0%	0%	0%	4%	4%
Splice Site	0%	33%	0%	0%	0%	0%	8%	0%
**ERBB2**								
Frame Shift Deletion	0%	8%	0%	0%	6%	0%	5%	4%
Frame Shift Insertion	0%	0%	0%	0%	6%	0%	0%	4%
In Frame Deletion	0%	0%	0%	0%	6%	0%	0%	0%
Missense Mutation	100%	92%	100%	100%	81%	100%	95%	90%
Splice Site	0%	0%	0%	0%	0%	0%	0%	2%
**ERBB3**								
Frame Shift Deletion	0%	0%	0%	0%	6%	0%	3%	7%
Frame Shift Insertion	0%	0%	0%	25%	0%	0%	3%	0%
In Frame Deletion	0%	0%	0%	0%	0%	0%	0%	5%
Missense Mutation	100%	100%	100%	75%	88%	95%	92%	85%
Nonsense Mutation	0%	0%	0%	0%	0%	5%	0%	2%
Splice Site	0%	0%	0%	0%	0%	0%	3%	2%
Translation Start Site	0%	0%	0%	0%	6%	0%	0%	0%
**ERBB4**								
Frame Shift Deletion	0%	6%	0%	33%	0%	4%	0%	4%
Frame Shift Insertion	0%	24%	0%	0%	6%	0%	2%	2%
Missense Mutation	100%	71%	100%	67%	78%	82%	89%	76%
Nonsense Mutation	0%	0%	0%	0%	11%	11%	7%	10%
Splice Site	0%	0%	0%	0%	6%	4%	2%	8%
**ERRFI1**								
Frame Shift Deletion	0%	0%	0%	0%	33%	0%	33%	50%
Frame Shift Insertion	0%	0%	0%	0%	0%	0%	17%	0%
Missense Mutation	0%	0%	0%	0%	33%	100%	50%	50%
Nonsense Mutation	0%	0%	0%	0%	33%	0%	0%	0%
**FGFR1**								
Frame Shift Deletion	0%	0%	0%	0%	0%	14%	0%	6%
Missense Mutation	0%	100%	0%	0%	90%	86%	94%	88%
Splice Site	0%	0%	0%	0%	10%	0%	6%	6%
**FGFR2**								
Frame Shift Deletion	0%	0%	0%	0%	20%	0%	11%	0%
Frame Shift Insertion	0%	0%	0%	0%	0%	0%	11%	0%
Missense Mutation	0%	75%	0%	100%	40%	80%	78%	85%
Nonsense Mutation	0%	25%	0%	0%	40%	20%	0%	8%
Splice Site	0%	0%	0%	0%	0%	0%	0%	8%
**FGFR3**								
Frame Shift Deletion	0%	0%	0%	0%	40%	0%	5%	10%
Frame Shift Insertion	0%	0%	0%	0%	0%	15%	0%	10%
Missense Mutation	100%	100%	100%	100%	60%	77%	95%	76%
Nonsense Mutation	0%	0%	0%	0%	0%	8%	0%	0%
Splice Site	0%	0%	0%	0%	0%	0%	0%	5%
**FGFR4**								
Frame Shift Deletion	100%	0%	0%	0%	17%	0%	0%	20%
Frame Shift Insertion	0%	0%	0%	0%	0%	0%	0%	4%
Missense Mutation	0%	100%	100%	100%	83%	100%	100%	72%
Splice Site	0%	0%	0%	0%	0%	0%	0%	4%
**FLT3**								
Frame Shift Deletion	0%	0%	0%	0%	0%	17%	0%	16%
Frame Shift Insertion	0%	0%	0%	67%	0%	6%	0%	0%
Missense Mutation	0%	100%	50%	33%	100%	67%	100%	74%
Nonsense Mutation	0%	0%	50%	0%	0%	6%	0%	0%
Splice Site	100%	0%	0%	0%	0%	6%	0%	11%
**HRAS**								
Missense Mutation	100%	100%	0%	100%	100%	100%	100%	100%
**IGF1R**								
Frame Shift Deletion	0%	0%	100%	0%	10%	0%	3%	7%
Frame Shift Insertion	0%	0%	0%	0%	10%	0%	3%	10%
Missense Mutation	100%	100%	0%	100%	70%	80%	90%	79%
Nonsense Mutation	0%	0%	0%	0%	0%	20%	0%	3%
Splice Site	0%	0%	0%	0%	10%	0%	3%	0%
**KIT**								
Frame Shift Insertion	0%	0%	0%	0%	0%	0%	14%	0%
Missense Mutation	100%	100%	100%	0%	100%	89%	86%	87%
Nonsense Mutation	0%	0%	0%	0%	0%	6%	0%	10%
Splice Site	0%	0%	0%	0%	0%	6%	0%	3%
**KRAS**								
Frame Shift Insertion	0%	0%	0%	0%	0%	0%	0%	0%
In Frame Insertion	0%	0%	0%	0%	0%	0%	1%	0%
Missense Mutation	100%	100%	100%	100%	99%	100%	99%	100%
Nonsense Mutation	0%	0%	0%	0%	1%	0%	0%	0%
**MAP2K1**								
Frame Shift Deletion	0%	0%	0%	0%	0%	0%	8%	20%
In Frame Deletion	0%	0%	0%	0%	0%	0%	0%	30%
Missense Mutation	100%	100%	0%	0%	100%	100%	92%	50%
**MAP2K2**								
Frame Shift Insertion	0%	0%	0%	0%	33%	0%	0%	0%
Missense Mutation	100%	100%	100%	100%	67%	100%	88%	100%
Splice Site	0%	0%	0%	0%	0%	0%	13%	0%
**MAPK1**								
In Frame Deletion	0%	0%	0%	0%	33%	0%	0%	17%
Missense Mutation	0%	100%	0%	0%	67%	100%	0%	83%
Nonsense Mutation	0%	0%	0%	0%	0%	0%	100%	0%
**MAPK3**								
In Frame Deletion	0%	0%	0%	0%	0%	0%	0%	20%
Missense Mutation	100%	100%	100%	100%	100%	100%	80%	80%
Nonsense Mutation	0%	0%	0%	0%	0%	0%	20%	0%
**MET**								
Frame Shift Deletion	0%	0%	20%	50%	0%	7%	14%	11%
Frame Shift Insertion	0%	0%	0%	0%	20%	0%	0%	4%
In Frame Insertion	0%	0%	0%	0%	0%	0%	14%	0%
Missense Mutation	100%	0%	80%	50%	80%	71%	64%	78%
Nonsense Mutation	0%	0%	0%	0%	0%	21%	7%	7%
**NF1**								
Frame Shift Deletion	25%	14%	33%	0%	5%	8%	9%	3%
Frame Shift Insertion	0%	9%	0%	0%	11%	15%	8%	5%
In Frame Insertion	0%	0%	0%	0%	0%	0%	0%	2%
Missense Mutation	50%	59%	33%	0%	47%	69%	57%	66%
Nonsense Mutation	25%	9%	0%	0%	32%	4%	15%	20%
Splice Site	0%	9%	33%	100%	5%	4%	11%	3%
**NRAS**								
Missense Mutation	100%	100%	100%	100%	100%	100%	100%	100%
**NTRK1**								
Frame Shift Deletion	0%	0%	0%	33%	0%	9%	0%	0%
Frame Shift Insertion	0%	0%	0%	0%	10%	0%	0%	0%
Missense Mutation	100%	100%	100%	67%	90%	91%	100%	81%
Nonsense Mutation	0%	0%	0%	0%	0%	0%	0%	19%
**NTRK2**								
Missense Mutation	100%	100%	0%	100%	100%	89%	100%	80%
Nonsense Mutation	0%	0%	100%	0%	0%	11%	0%	20%
**PDGFRA**								
Frame Shift Deletion	0%	0%	0%	0%	6%	0%	0%	0%
Frame Shift Insertion	0%	0%	0%	0%	0%	0%	5%	3%
Missense Mutation	100%	100%	0%	0%	81%	81%	89%	73%
Nonsense Mutation	0%	0%	0%	0%	6%	11%	5%	17%
Splice Site	0%	0%	0%	0%	6%	7%	0%	7%
**PTPN11**								
Frame Shift Deletion	0%	0%	33%	0%	0%	0%	0%	0%
Frame Shift Insertion	0%	0%	0%	0%	20%	0%	0%	0%
Missense Mutation	0%	0%	67%	0%	80%	100%	78%	100%
Nonsense Mutation	0%	0%	0%	100%	0%	0%	11%	0%
Splice Region	0%	0%	0%	0%	0%	0%	11%	0%
**RAC1**								
Missense Mutation	0%	0%	0%	0%	100%	100%	0%	100%
**RAF1**								
Frame Shift Deletion	0%	0%	50%	0%	0%	0%	0%	10%
Frame Shift Insertion	0%	0%	0%	0%	0%	0%	13%	5%
Missense Mutation	100%	100%	50%	100%	89%	89%	75%	81%
Nonsense Mutation	0%	0%	0%	0%	11%	11%	0%	5%
Splice Site	0%	0%	0%	0%	0%	0%	13%	0%
**RASA1**								
Frame Shift Deletion	100%	50%	50%	0%	0%	5%	17%	15%
Frame Shift Insertion	0%	0%	25%	0%	8%	0%	9%	6%
In Frame Deletion	0%	0%	0%	0%	0%	0%	0%	3%
Missense Mutation	0%	25%	25%	0%	62%	71%	57%	62%
Nonsense Mutation	0%	25%	0%	0%	31%	14%	13%	9%
Splice Site	0%	0%	0%	100%	0%	10%	4%	6%
**RET**								
Frame Shift Deletion	0%	0%	0%	0%	13%	0%	0%	0%
Missense Mutation	50%	0%	100%	50%	88%	100%	100%	100%
Nonsense Mutation	50%	0%	0%	0%	0%	0%	0%	0%
Splice Site	0%	100%	0%	50%	0%	0%	0%	0%
**RIT1**								
Frame Shift Insertion	0%	0%	0%	0%	0%	0%	17%	0%
Missense Mutation	100%	100%	100%	0%	100%	67%	50%	80%
Nonsense Mutation	0%	0%	0%	0%	0%	17%	33%	0%
Splice Site	0%	0%	0%	0%	0%	17%	0%	20%
**ROS1**								
Frame Shift Deletion	0%	0%	0%	0%	5%	8%	6%	10%
Frame Shift Insertion	0%	0%	0%	0%	5%	0%	3%	7%
Missense Mutation	100%	75%	83%	50%	90%	88%	81%	68%
Nonsense Mutation	0%	25%	17%	0%	0%	4%	8%	12%
Splice Site	0%	0%	0%	50%	0%	0%	3%	2%
**SOS1**								
Frame Shift Deletion	0%	0%	0%	0%	11%	8%	25%	7%
Frame Shift Insertion	0%	0%	0%	0%	0%	0%	0%	7%
Missense Mutation	0%	0%	100%	0%	67%	75%	75%	80%
Nonsense Mutation	0%	100%	0%	0%	22%	17%	0%	7%

Table S14. Distribution of Colorectal Cancer Cases by Data Source, Ancestry, Age at Onset, and FOLFOX Treatment Status. This table summarizes the number and proportion of colorectal cancer cases contributing to the Hispanic/Latino and non-Hispanic White cohorts across the MSK-CHORD, TCGA Pan-Cancer Atlas, and AACR Project GENIE databases. Cases are stratified by age at diagnosis (early-onset <50 years vs. late-onset ≥50 years) and FOLFOX chemotherapy exposure. The table highlights the relative contribution of each data source to ancestry-, age-, and treatment-defined subgroups, providing transparency regarding cohort composition and supporting the rationale for analytic decisions related to normalization and batch-effect considerations.

	**Hispanic/Latino Cohort n (%)**			**Non-Hispanic White Cohort n (%)**	
**Clinical Feature**	**MSK CHORD**	**TCGA PanCancer Atlas**	**AACR Project Genie**	**MSK CHORD**	**TCGA PanCancer Atlas**
**Age Onset & Treatment**					
Early-Onset (< 50) Treated with FOLFOX	57 (24.5%)	1 (50.0%)	15 (48.4%)	374 (16.7%)	1 (12.5%)
Late-Onset (≥ 50) Treated with FOLFOX	80 (34.3%)	1 (50.0%)	10 (32.3%)	916 (40.9%)	3 (37.5%)
Early-Onset (< 50) Not Treated with FOLFOX	48 (20.6%)	0 (0.0%)	4 (12.9%)	301 (13.4%)	1 (12.5%)
Late-Onset (≥ 50) Not Treated with FOLFOX	48 (20.6%)	0 (0.0%)	2 (6.5%)	650 (29.0%)	3 (37.5%)

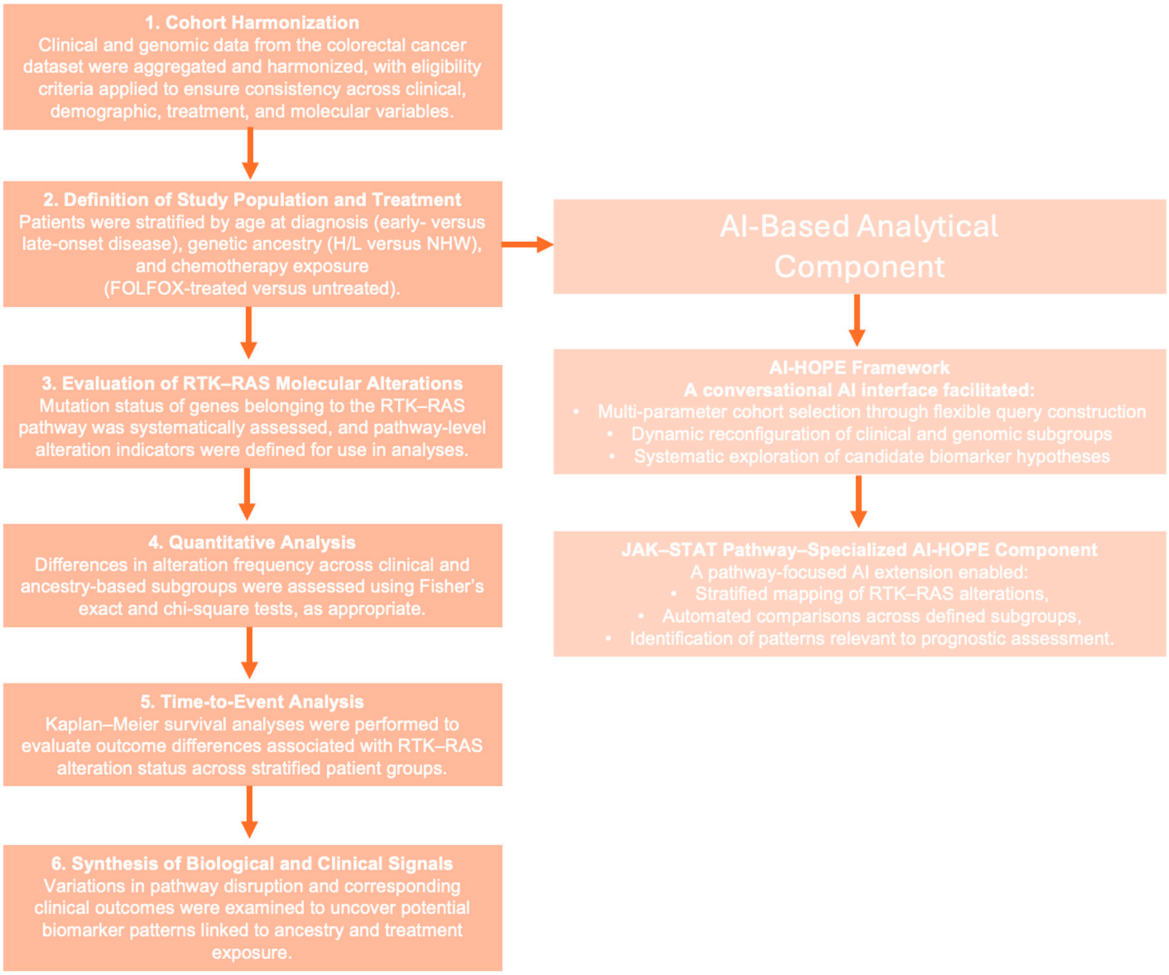

Figure S1. Schematic Overview of the AI-HOPE–Enabled Analytical Workflow for RTK–RAS Biomarker Discovery. This schematic illustrates the stepwise AI-HOPE–enabled analytical pipeline used in this study. Clinical and genomic data from colorectal cancer cohorts were first aggregated and harmonized with standardized eligibility criteria. Patients were then stratified by age at diagnosis, ancestry, and FOLFOX treatment exposure. RTK–RAS pathway alterations were systematically evaluated at the gene and pathway levels, followed by statistical comparisons of alteration frequencies across clinically and ancestry defined subgroups. Survival outcomes were assessed using Kaplan-Meier analyses to evaluate the prognostic impact of RTK-RAS alterations. The AI-based analytical component integrates the AI-HOPE conversational framework and the pathway-specialized AI-HOPE modules to enable multi-parameter cohort filtering, dynamic subgroup reorganization, automated comparisons, and rapid identification of biologically and clinically relevant patterns, supporting scalable and hypothesis-driven precision oncology analyses.

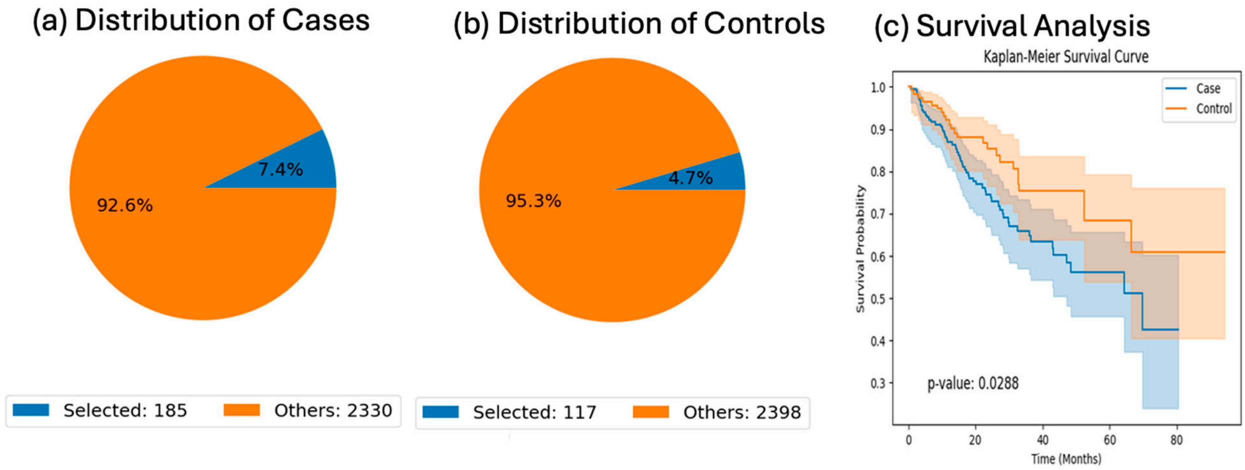

Figure S2. AI-enabled cohort definition and survival stratification of early-onset colorectal cancer (EOCRC) non-Hispanic White (NHW) patients not treated with FOLFOX based on RTK-RAS pathway status. AI-HOPE was used to programmatically define case and control cohorts using integrated clinical variables (ancestry, age at onset, treatment exposure) and pathway-level genomic annotations. The case cohort consisted of EOCRC NHW patients who did not receive FOLFOX and harbored at least one RTK-RAS pathway alteration (n = 185), while the control cohort included EOCRC NHW patients without RTK-RAS alterations and no FOLFOX exposure (n = 117). Pie charts illustrate the proportion of selected samples relative to the full dataset for each cohort. Kaplan-Meier analysis demonstrates a significant difference in overall survival between altered and non-altered groups, with RTK-RAS-altered tumors exhibiting inferior survival outcomes (log-rank p = 0.0288). Shaded regions represent 95% confidence intervals, highlighting increased early divergence of survival probabilities between groups.

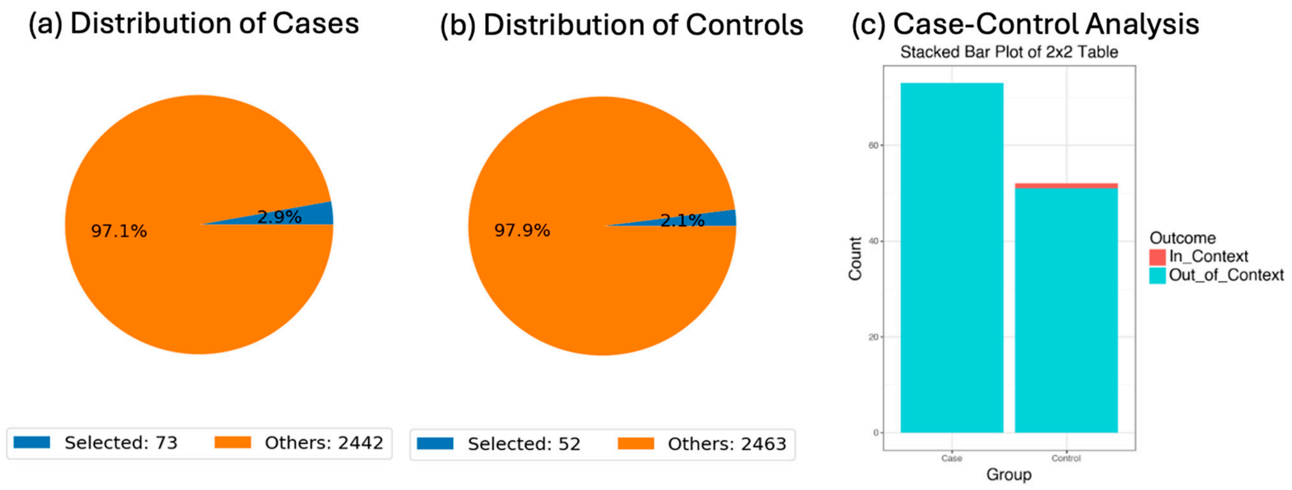

Figure S3. AI-driven case-control composition and comparative analysis of pathway-defined cohorts in early-onset colorectal cancer (EOCRC). This figure summarizes the AI-HOPE-guided construction and evaluation of case and control cohorts derived from EOCRC patients under predefined clinical and genomic constraints. Panel (a) illustrates the proportion of samples meeting the case criteria (selected/in-context) relative to the full dataset, while panel (b) shows the corresponding distribution for the control cohort. In both groups, selected samples represent a small, highly specific subset of the overall population, reflecting stringent cohort definition. Panel (c) presents a stacked bar visualization of the resulting 2×2 case-control table, comparing in-context versus out-of-context samples across groups. Statistical assessment was performed using Fisher's exact testing to evaluate differences between cohorts, demonstrating no significant enrichment of the queried genomic feature between cases and controls. Together, these plots highlight the transparency of AI-assisted cohort selection and provide a quantitative framework for downstream comparative analyses.

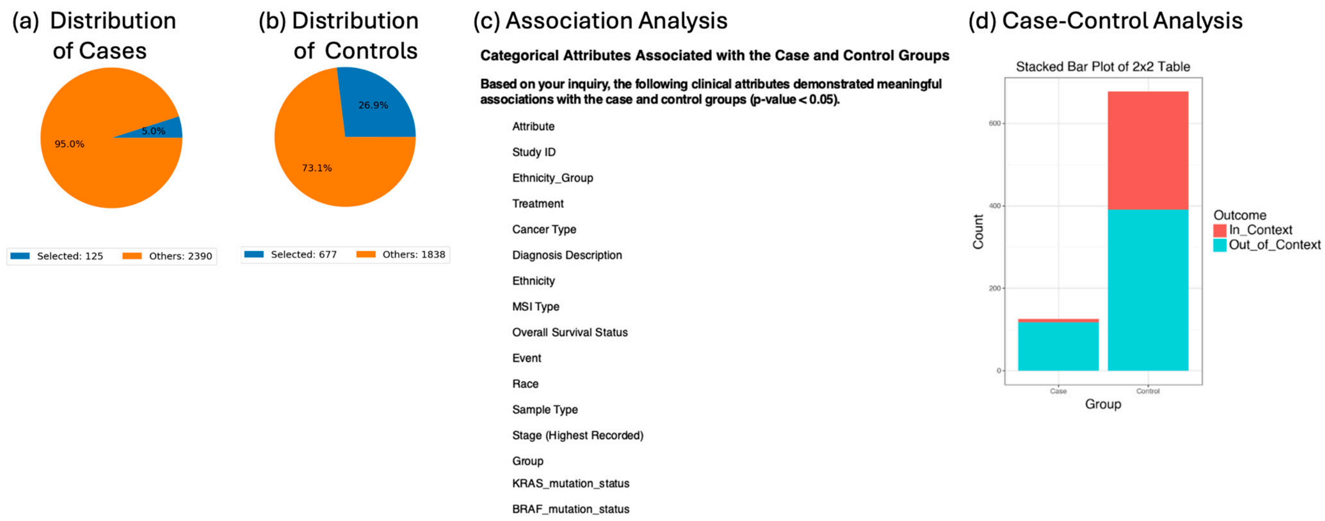

Figure S4. Systematic AI-assisted comparison of clinical and genomic features between early-onset colorectal cancer (EOCRC) Hispanic/Latino (H/L) and Non-Hispanic White (NHW) cohorts. This figure summarizes the output of an AI-HOPE-driven exploratory analysis designed to identify clinical and molecular attributes that distinguish EOCRC cases by ancestry. a) The case cohort comprised EOCRC H/L patients (n = 125), b) while the control cohort included EOCRC NHW patients (n = 677), both defined using uniform age-of-onset criteria. Pie charts depict the relative proportions of samples meeting the user-defined analytic context versus the remaining dataset for each cohort, illustrating the differential enrichment of in-context samples across ancestries. c) The accompanying table enumerates categorical variables demonstrating statistically significant associations between case and control groups ($p < 0.05$), spanning demographic descriptors (ethnicity and race), diagnostic and tumor characteristics (cancer type, diagnosis description, sample type, and stage at diagnosis), clinical outcomes (overall survival status and event), and recurrent oncogenic alterations, including SMAD4, APC, TP53, and KRAS mutation status. d) The stacked bar plot provides a visual summary of the 2×2 comparison underlying the odds-ratio framework used to assess group-level enrichment. These results underscore ancestry-associated heterogeneity across multiple clinical and molecular dimensions in EOCRC

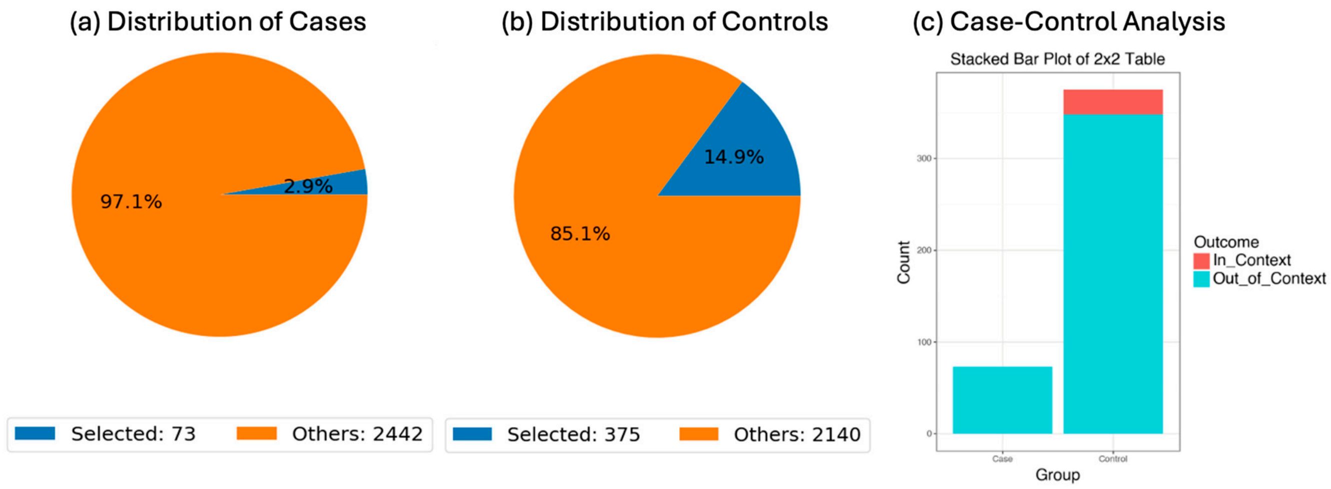

Figure S5. AI-assisted case-control evaluation of BRAF mutation prevalence in FOLFOX-treated EOCRC by ancestry. This figure illustrates an AI-HOPE-guided comparison of BRAF mutation status between EOCRC Hispanic/Latino (H/L) and Non-Hispanic White (NHW) colorectal cancer (CRC) patients who received FOLFOX chemotherapy. The case cohort comprises EOCRC H/L patients (n = 73), while the control cohort includes EOCRC NHW patients (n = 375). (a) and (b) Pie charts depict the proportion of in-context (BRAF-mutant) and out-of-context samples within the case and control cohorts, respectively, highlighting differences in BRAF mutation representation across ancestries. (c) A stacked bar plot summarizes the corresponding 2×2 contingency table used for odds-ratio testing, contrasting BRAF-mutant and wild-type samples between groups. Statistical assessment was performed using Fisher's exact test, with results indicating a differential distribution of BRAF mutations between EOCRC H/L and NHW patients treated with FOLFOX. Together, these analyses support ancestry-associated variation in RTK-RAS pathway alterations within this clinically relevant treatment subgroup.
